# ReBiA—Robotic Enabled Biological Automation: 3D Epithelial Tissue Production

**DOI:** 10.1002/advs.202406608

**Published:** 2024-09-26

**Authors:** Lukas Königer, Christoph Malkmus, Dalia Mahdy, Thomas Däullary, Susanna Götz, Thomas Schwarz, Marius Gensler, Niklas Pallmann, Danjouma Cheufou, Andreas Rosenwald, Marc Möllmann, Dieter Groneberg, Christina Popp, Florian Groeber‐Becker, Maria Steinke, Jan Hansmann

**Affiliations:** ^1^ Translational Center Regenerative Therapies Fraunhofer Institute for Silicate Research ISC 97070 Würzburg Germany; ^2^ Institute of Medical Engineering Schweinfurt Technical University of Applied Sciences Würzburg‐Schweinfurt 97421 Schweinfurt Germany; ^3^ Chair of Tissue Engineering and Regenerative Medicine University Hospital Würzburg 97070 Würzburg Germany; ^4^ Chair of Cellular Immunotherapy University Hospital Würzburg 97080 Würzburg Germany; ^5^ Faculty of Design Würzburg Technical University of Applied Sciences Würzburg‐Schweinfurt 97070 Würzburg Germany; ^6^ Department of Thoracic Surgery Klinikum Würzburg Mitte 97070 Würzburg Germany; ^7^ Institute of Pathology University of Würzburg 97080 Würzburg Germany; ^8^ Department of Ophthalmology University Clinic Düsseldorf 40225 Düsseldorf Germany; ^9^ Department of Oto‐Rhino‐Laryngology Plastic Aesthetic and Reconstructive Head and Neck Surgery University Hospital Würzburg 97080 Würzburg Germany

**Keywords:** 3R, AI tools, alternatives to animal testing, lab automation, tissue engineering

## Abstract

The Food and Drug Administration's recent decision to eliminate mandatory animal testing for drug approval marks a significant shift to alternative methods. Similarly, the European Parliament is advocating for a faster transition, reflecting public preference for animal‐free research practices. In vitro tissue models are increasingly recognized as valuable tools for regulatory assessments before clinical trials, in line with the 3R principles (Replace, Reduce, Refine). Despite their potential, barriers such as the need for standardization, availability, and cost hinder their widespread adoption. To address these challenges, the Robotic Enabled Biological Automation (ReBiA) system is developed. This system uses a dual‐arm robot capable of standardizing laboratory processes within a closed automated environment, translating manual processes into automated ones. This reduces the need for process‐specific developments, making in vitro tissue models more consistent and cost‐effective. ReBiA's performance is demonstrated through producing human reconstructed epidermis, human airway epithelial models, and human intestinal organoids. Analyses confirm that these models match the morphology and protein expression of manually prepared and native tissues, with similar cell viability. These successes highlight ReBiA's potential to lower barriers to broader adoption of in vitro tissue models, supporting a shift toward more ethical and advanced research methods.

## Graphical abstract

In vitro tissue models are a valuable tool for regulatory evaluation of drug candidates prior to clinical trials, but challenges such as standardization and cost hinder their adoption. The ReBiA system uses a dual‐arm robot to automate laboratory processes, ensuring consistent quality of tissue models and less costs. As a result, automated production supports the reduction of animal testing.

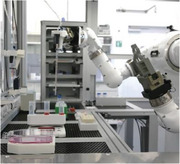

## 1 Introduction

The price of novel therapies and the development of a drug from bench to bedside is largely determined by the cost of the clinical phases, in which drug candidates are administered to humans.^[^ ^1^ ^]^ On average, only one in ten drug candidates passes clinical trials, despite having shown promising results in the preclinical phase, due to the low predictive power of applied preclinical test methods.^[^ ^1^, ^2^ ^]^ A strategy to reduce the discrepancy between the preclinical and clinical outcomes of drug evaluation is to use test systems with improved predictive power in research.

Animal testing is commonly used for efficacy and safety testing to eliminate drug candidates from further consideration in the preclinical phase,^[^ ^3^ ^]^ thereby advancing drug discovery.^[^ ^4^ ^]^ However, it is widely recognized that animal studies often do not provide translational accuracy in predicting drug effects on humans.^[^ ^3^, ^5^ ^]^ This is due to anatomical and physiological differences between animals and humans.^[^ ^6^ ^]^ It has also been reported that reliance on animal models limits the ability to learn and understand human diseases,^[^ ^7^ ^]^ e.g., the study of obligate human diseases such as pertussis or river blindness,^[^ ^8^, ^9^ ^]^ or the epithelial host response during infection with the gastroenteritis‐causing pathogen Salmonella Typhimurium. The latter has been shown to be different in human primary cell‐based models compared to previously reported animal studies.^[^ ^10^ ^]^ These scientific challenges, regulatory aspects as well as major public opinion contribute to the 3R principles—Reduction, Refinement, and Replacement of animal testing.^[^ ^11^ ^]^

As a complement to in vivo animal models, 2D cell cultures allow the evaluation of drugs and their impact on human cells in vitro.^[^ ^12^, ^13^ ^]^ However, a monolayer of human cells does not resemble the complexity and functionality of a tissue or an entire organ. Major differences include the lack of 3D structures including a circulatory system, a limited variety of cells, and the absence of an extracellular matrix. Thus, essential tissue functions are not represented in 2D cell cultures.^[^ ^13^, ^14^ ^]^ To overcome these drawbacks, tissue engineering focuses on extending cell culture into the third dimension. This increased complexity mimics the in vivo situation more closely, thereby strengthening the predictive power of 3D in vitro tissue models compared to conventional 2D cell culture.^[^ ^12^, ^14^ ^]^ A variety of 3D in vitro model systems have been established for multiple human tissues.^[^ ^15^ ^]^ Barrier tissues are of particular interest, as their protective function is essential for human health. For example, the human epidermis—the outermost layer of the skin—can be resembled by the in vitro reconstructed human epidermis (RHE), a tissue model consisting of isolated human epidermal keratinocytes (hEK). The wide range of applications of the RHE is demonstrated by regulatory accepted test guidelines to assess skin irritation and skin corrosion.^[^ ^16^ ^]^ In addition, various test methods based on skin tissue models are applied in basic research to study the formation of melanoma,^[^ ^17^ ^]^ pigmentation,^[^ ^18^ ^]^ psoriasis,^[^ ^19^ ^]^ atopic dermatitis,^[^ ^20^ ^]^ mechanisms of epidermal wound healing,^[^ ^21^ ^]^ or percutaneous parasite invasion.^[^ ^22^ ^]^ Another well‐established type of in vitro barrier tissue is airway epithelial models. The ciliated airways are the first line of defense against airborne pathogens. Human airway tissue models (hATM) are suitable to study host‐pathogen interactions with airborne bacteria or viruses, such as severe acute respiratory syndrome coronavirus type 2 (SARS‐CoV‐2), but also to understand respiratory diseases, such as cystic fibrosis or primary ciliary dyskinesia.^[^ ^8^, ^23^, ^24^, ^25^, ^26^ ^]^ Additionally, the hATM serves as a test system platform for inhalation toxicity.^[^ ^27^ ^]^

Despite their advantages and successful application for alternative testing, we identified barriers that impede the broad application of hATM and RHE for regulatory in vitro testing. There are strict requirements for the reproducibility and predictive power of 3D in vitro test systems in the regulatory context of efficacy and safety testing. Therefore, the Organization for Economic Cooperation and Development (OECD) has established regulatory guidelines that define criteria for testing procedures using tissue models.^[^ ^16^, ^28^ ^]^ Inaccuracies in the execution of the standard operating procedures (SOPs) and the expertise of the operators can lead to considerable variation, resulting in validation failure (**Barrier 1 – Regulatory Acceptance**). Tissue engineering processes are highly sensitive to minimal variations in factors such as incubation times, isolation of human primary cells, composition of prepared cell culture media, and individual operator practices. Therefore, an appropriate level of standardization is required to ensure the reproducibility of the in vitro tissues and analytic readouts (**Barrier 2 – Reproducibility**). In addition, the cell culture methodologies for tissue models are more demanding in terms of complex culture media, reagents, and handling compared to 2D cell culture. Such time‐intensive procedures increase the costs for 3D in vitro tissues (**Barrier 3 – Cost**). Finally, a sufficient availability of models of consistent quality is a prerequisite for their successful application (**Barrier 4 – Availability**).

These barriers are not specific to in vitro test systems and have already been overcome in various industries by automation to counteract rising production costs, high standard deviations, and regulatory hurdles. Automation in life sciences mainly covers repetitive and labor‐intensive analytical processes and process steps, such as precise liquid handling of ELISA and PCR‐based analyses,^[^ ^29^, ^30^ ^]^ thereby reducing material and personnel costs. Another option for automated liquid handling is pipetting robots^[^ ^31^, ^32^, ^33^ ^]^ that allow standardization of critical dosing and handling of different substances, thus minimizing the operator's influence on the process. In tissue engineering, the fabrication, cultivation, and screening of 3D cancer constructs in alginate beads could be automated.^[^ ^34^, ^35^ ^]^ However, the robotic platform using a single‐arm robot shown by Lehmann et al. is limited in the flexibility due to specific liquid handling units **(Barrier 5 – Flexibility)**. Single‐arm robots were also shown to be applied for in situ bioprinting for biomedical research and chemical synthesis assisted by artificial intelligence (AI) planning.^[^ ^36^, ^37^ ^]^ Mobile robots can already assist researchers as demonstrated in the improvement of photocatalyst mixtures.^[^ ^38^ ^]^ To enable more complex handling, dual‐arm robots have found their way into the life sciences in recent years. Publications have already demonstrated that the dual‐arm concept promotes the use of generic laboratory materials in an automated context. This has been shown for biological sample preparation and analysis^[^ ^39^ ^]^ as well as for the automated maintenance of 2D cell cultures by the LabDroid Maholo.^[^ ^40^, ^41^ ^]^ More recently, Hamm et al. demonstrated the feasibility of automated 2D cell culture with modified generic materials using a single arm robot.^[^ ^42^ ^]^ To our knowledge, a fully automated process for 3D tissue model systems has not yet been presented.

To overcome barriers 1–5, the construction processes for 3D tissue models need to be fully automated, thereby enabling the adoption of alternative test systems as a new standard across both fundamental research and industrial applications. The aim of this study was to design a robotic platform for cell culture and tissue engineering applications. Instead of developing highly specialized solutions for individual process steps, a flexible and holistic approach involving the use of generic laboratory materials was envisioned to automate the RHE and hATM production processes.

## 2 Materials and Methods

### 2.1 Primary Human Cell Culture

#### 2.1.1 Donor Information and Cell Isolation

Primary human epidermal keratinocytes (hEK) were isolated from juvenile prepuce biopsies of donors aged between 2 and 5 years, according to ethical approval granted by the institutional ethics committee of the Julius‐Maximilians‐University Würzburg (vote 182/10 and 280/18) and under‐informed consent by the legal guardians of the donors. The cell isolation was performed as previously published.^[^ ^43^ ^]^ Human airway tissue specimens were obtained from eight male and five female donors (18–72 years old) for isolation of airway epithelial cells (hAEC) and fibroblasts (hAF). Written informed consent was obtained beforehand and the studies were approved by the institutional ethics committees on human research of the Julius‐Maximilians‐University Würzburg (votes 99/20, 179/17, and 116/17) and Otto‐von‐Guericke University Magdeburg (vote 163/17), respectively. Airway cells were isolated according to previously published protocols.^[^ ^8^, ^44^, ^45^ ^]^ During airway cell isolation, tiny parts of the tissue samples were put on glass carriers and observed under a light microscope. Epithelial cells with ciliary beating, which indicated that viable and functional tissue samples were used for airway tissue model construction (Video S1, Supporting Information) were identified.

#### 2.1.2 Cell Culture Media

hEK were cultured in EpiLife medium (MEPI‐500CA; Thermo Fisher Scientific, USA) supplemented with factors supporting the maintenance and differentiation in each specific culture phase. EpiLife media were successively supplemented: E1 – 2D culture: 1× Penicillin/Streptomycin (Pen/Strep; P4333; Sigma–Aldrich, USA), 1× Human keratinocyte growth supplement (S0015, Thermo Fisher Scientific, USA). E2 – RHE setup: 1.44 mm CaCl_2_ (C7902; Sigma–Aldrich, USA). E3 – 3D culture: 252 µm ascorbic acid (A8960; Sigma–Aldrich, USA), 10 ng mL^−1^ Keratinocyte growth factor (K1757; Sigma–Aldrich, USA).

hAEC were grown in Airway Epithelial Cell Growth Medium (AECG; PB‐C‐MH‐350‐0099; PeloBiotech, Germany). hAF were grown in DMEM (61965‐026; Thermo Fisher Scientific, USA) supplemented with 10% fetal bovine serum (P30‐3306; PAN biotech, Germany). For cell co‐culture, a culture medium mixture of AECG and fibroblast culture medium supplemented with 1× Pen/Strep was used.

Human small intestinal organoids (hIO) were grown in human spheroid maintenance medium (hSM) as previously described.^[^ ^10^, ^46^, ^47^, ^48^ ^]^ Briefly, hSM consisted of 75% conditioned L‐WRN medium (contains Wnt‐3A, R‐Spondin‐1, and Noggin, produced by cell‐line L‐WRN (CRL‐3276; ATCC, USA))^[^ ^49^ ^]^ combined with 25% Advanced DMEM F12 (12634‐028; Thermo Fisher Scientific, USA) with 10 mm HEPES (H3662; Sigma Aldrich, USA), 1× GlutaMax‐I (35050‐061; Thermo Fisher Scientific, USA), 1× Anti‐Anti (15240‐062; Thermo Fisher Scientific, USA), 1 mm N‐Acetylcysteine (A9165; Sigma–Aldrich, USA), 1× N2‐Supplement (17502‐048; Thermo Fisher Scientific, USA), 1× B27‐Supplement without vitamin A (12587‐010; Thermo Fisher Scientific, USA) complemented with 0.05 µg mL^−1^ mEGF (AF‐100‐15; Peprotech, Germany), 0.01 µm Leu‐Gastrin (G9145; Sigma–Aldrich, USA), 10 µm Nicotinamide (N0636; Sigma Aldrich, USA), 0.5 µm A83–01 (2939; Tocris Bioscience, Germany), 10 µm SB202190 (S7067; Sigma Aldrich, USA), 0.5 µm LY2157299 (CAY15312; CAYMAN Chemical Company, UK).

#### 2.1.3 2D Cell Culture

For expansion, hEK were cultured in cell culture flasks using E1 medium. The medium was changed three times per week and cells were incubated under standard conditions (37 °C, 5% CO_2_, 95% humidity). hEK were cultured until passage 2 and after cryopreservation used for the RHE generation, in which the cells were in passage 3. Confluence was kept under 90%. Primary airway cells were cultured under standard conditions and the medium was changed three times per week. To build the tissue models, hAEC were used in passage 1 and hAF were used in passages 1–6.

#### 2.1.4 Human Organoid Culture

Human intestinal organoids (hIOs) were cultured as previously described.^[^ ^10^, ^46^, ^47^, ^48^ ^]^ Briefly, prior automated cell seeding hIOs were dissociated enzymatically and mechanically into single cells, followed by resuspension in ice‐cold Matrigel (354230; Corning, USA) with a density of 1000 single cells µL^−1^. Single cells were applied in 5–20 µL droplets into pre‐warmed 24‐well plates. After solidification of the Matrigel droplets at 37 °C, the droplets were covered with 1.5 mL hSM and incubated for up to 6 days under standard conditions (37 °C, 5% CO_2_, 95 % humidity).

#### 2.1.5 Manual Generation of RHE

The RHE models were set up as previously published.^[^ ^50^ ^]^ Briefly, hEKs were detached by accutase (A1110501; Thermo Fisher Scientific, USA). After cell counting and adjustment of cell concentration—3 × 10^5^ hEK in 200 µL E2 medium were seeded into Millicell cell culture inserts (PIHP01250; Merck Millipore, Germany). After 2 h, the cells adhered to the membrane, followed by the addition of E2 medium into the surrounding well/compartment—1.5 mL per well for manually manufactured models on six‐well plates. After 24 h, the models were set to the air–liquid‐interface (ALI) by aspiration of the submerging medium, and the surrounding E2 medium was replaced by E3 for the RHE culture phase. The medium was replaced three times per week and the tissue models were cultured under standard conditions for 19 days.

#### 2.1.6 Manual Generation of hATM

As a 3D scaffold for tissue model generation, decellularized segments of the porcine small intestinal submucosa (SIS) were used, as previously described.^[^ ^8^, ^44^, ^45^ ^]^ Animal research was performed according to German law and institutional guidelines approved by the Ethics Committee of the District of Unterfranken, Würzburg, Germany (approval number 55.2‐2532‐2‐256). Each scaffold was seeded with 2.5 × 10^4^ hAF from the apical side and cultured under submerged conditions. The next day, 1.25 × 10^5^ hAEC were added from the apical side, and the whole construct was submerged for 24–48 h. Then, the primary tissue models matured at the ALI for 3 weeks until beating kinocilia and mucus production were observed at the light microscopic level (Video S2, Supporting Information). Video recording was done using an ECLIPSE Ci‐L microscope (Nikon, Japan) and a high‐speed camera (Motion Traveller 1000; Imaging Solutions, Germany). For cell co‐culture, a mixture made of 50% AECG and 50% hAF medium was used. Manual and automated construction of human airway tissue models was performed in parallel using cells derived from the same tissue specimen and under comparable experimental conditions. hATM were cultured for 21 days.

#### 2.1.7 Metabolite Analysis

Culture medium analysis was performed using Cedex BioAnalyzer (06395554001; Roche, Switzerland). 500 µL of the cell culture medium was taken before the regular medium exchange was performed. The concentration of glucose, lactate, and lactate dehydrogenase (LDH) was quantified using the respective kits (06343732001, 06343759001, 06343767001; Roche, Switzerland) according to the company's protocol.

#### 2.1.8 Assessment of the Barrier Functionality

Transepithelial electrical resistance (TEER) was measured according to a previously published protocol:^[^ ^21^ ^]^ A custom measurement system was used to analyze the TEER values of the RHE models. Therefore, EpiLife medium supplemented with 1% Pen/Strep and 1.44 mm CaCl_2_ was used as the liquid phase. A custom automated setup was used to generate an electrical frequency ranging from 1 Hz to 100 kHz at 0.2 V limited to 3 mA, as already described.^[^ ^51^ ^]^

#### 2.1.9 Cellular Metabolic Activity Assay

The viability of the RHE models was verified by investigating the metabolic activity of the cells. This was examined by performing a 3‐(4,5‐dimethylthiazol‐2‐yl)‐2,5‐diphenyltetrazoliumbromid (MTT) assay. On day 19, the RHE models were transferred into a 24‐well plate filled with 200 µL MTT solution (1 mg mL^−1^; 20395.03; SERVA Electrophoresis, Germany) in each well. After incubation for 3 h at 37 °C, each model was transferred into wells containing 2 mL isopropanol (9781.1; Carl Roth, Germany). After incubation overnight at 4 °C, the formazan was dissolved. For each RHE model, duplicates of 200 µL were used to measure the optical absorbance at 570 nm using the nfinite M200 plate reader (TECAN Trading, Switzerland).

For the comparative analysis of manually and automatically generated hATM during differentiation, qualitative and quantitative MTT assays were performed at three different time points (weeks 1, 2, and 3 after ALI introduction). The models were treated with the MTT solution as already described. After the MTT solution was removed, the models were washed once with PBS^−^ (Sigma–Aldrich, USA) and photographed for qualitative analysis. For the quantitative MTT analysis, the models were covered stepwise with 4 or 6 mL isopropanol and incubated for up to 3 h until clearance. The mean absorbance was measured as previously described and the values of the three independent experiments (*n = 3*, two models per time point).

The viability and distribution of hIO were assessed by MTT. For that, Matrigel droplets containing hIO were carefully washed with PBS^−^ without detaching the droplet, followed by a qualitative and quantitative MTT assay. MTT solution was applied and incubated for 3 h at 37 °C. For qualitative analysis, images were recorded. For quantitative analysis, the droplets were detached and incubated with isopropanol overnight at 4 °C, until the formazan was dissolved. Subsequently, the mean absorbance at 570 nm was measured as previously described.

#### 2.1.10 Skin Irritation Test Adapted from OECD 439

The skin irritation assay was manually conducted based on the OECD guideline 439.^[^ ^16^ ^]^ This guideline outlines a standardized method for in vitro testing of skin irritation, providing an alternative to in vivo skin irritation tests.

RHE models were automatically cultured until full differentiation on day 19 as described. The test substances were apically applied to the RHE model. The application volume was set to 25 µL and exposure time to 35 ± 5 min. After the exposure period, washing was performed in several steps. First, each model was thoroughly rinsed with eight times 600 µL PBS^+^, then immersed three times in a beaker of PBS^+^ and emptied, and repeated twice. Finally, the PBS^+^ was carefully aspirated both internally and externally. This was followed by a recovery period, where the RHE models were incubated for 42 h. The main endpoint of the OECD 439 guideline is tissue viability, assessed using the MTT assay. The results were expressed as a percentage of viability compared to negative controls (untreated RHE models). Chemicals reducing cell viability below a threshold of 50% were classified as irritants (cat. 2). For every experiment, three replicates per test substance and control were used. All substances were prepared at specified concentrations and applied in liquid form. For positive control, SDS was used at a concentration of 5%, while PBS^+^ was used as a negative control. Chemicals listed in **Table** **1** were tested for their potential to cause skin irritation.

**Table 1:** Irritation substances.

Chemical	Chemical abstract service registry number (CAS‐Nr.)
Isopropanol	67‐63‐0
Piperonyl butoxide	51‐03‐6
Heptanal	111‐71‐7
1‐Bromohexane	111‐25‐1

#### 2.1.11 Histological Analyses

For the histological analyses, RHE and hATM were fixed in a 4% paraformaldehyde solution (P087.1; Carl Roth, Germany). After paraffin embedding and micro‐sectioning, Hematoxylin and Eosin staining (HE) were performed according to a standard protocol. For the analysis of epidermis‐specific marker expression, tissue sections were immunohistochemically stained for Cytokeratin 10 (CK10; 1:100; M7002; Dako, USA) and 14 (CK14; 1:1000; HPA023040; Sigma–Aldrich, USA). For immunofluorescent staining of airway tissue models, the following primary antibodies were used: monoclonal rabbit anti‐Cytokeratin 18 (CK18; 1:100; NBP2‐67370; Novus Biologicals, USA), monoclonal rabbit anti‐vimentin (VIM; 1:1000; ab92547; Abcam, UK), and monoclonal mouse anti‐acetylated tubulin (1:1000; T7451; Sigma–Aldrich, USA). Primary antibodies were incubated overnight at 4 °C and secondary antibodies were incubated at room temperature for 2 h. For primary antibody detection, polyclonal donkey anti‐rabbit antibodies coupled to Alexa Fluor 488 (1:400; A‐21206; Thermo Fisher Scientific, USA) and polyclonal donkey anti‐rabbit antibodies coupled to Alexa Fluor 555 (1:400; A‐31572; Thermo Fisher Scientific, USA) were used, respectively. Sections were mounted with DAPI Fluoromount‐G (SBA‐0100‐20; Biozol, Germany) and images were taken with the BZ‐9000 BIOREVO System (Keyence, Germany). For whole‐mount staining, airway tissue models were prepared as reported previously^[^ ^8^ ^]^ using primary antibodies against ZO‐1 (1:1000; 21773‐1‐AP; ProteinTech, UK) to visualize tight junctions. Images and videos were made with a LEICA SP8 confocal laser scanning microscope and processed with LAS‐X software (Leica Microsystems, Germany).

#### 2.1.12 Sterilization Methods

Autoclaving of the Polydimethylsiloxane (PDMS)‐casted positioning plate was performed at 121 °C for 15 min using a DX‐45 autoclaving device (Systec, Germany). The 3D‐printed holding plates were sterilized by vaporized hydrogen peroxide (H_2_O_2_) plasma sterilization as previously published.^[^ ^52^ ^]^ Briefly, oxygen plasma was induced in a plasma cleaner device (Pico LF PC 115656; Diener electronic & Co. KG, Germany) to preheat the chamber (500 W, 12 min, 0.3 mbar, 12 sccm (standard cubic centimeters per minute)). The foil‐wrapped (Stericlin; VP Group, Germany) parts were placed into the chamber together with a vaporizer unit filled with 1.5 mL of 60% H_2_O_2_‐solution (10746291; Thermo Fisher Scientific, USA). This way, the parts were incubated for 75 min at 4 mbar, which generated an H_2_O_2_ atmosphere. Following, the parts were plasma treated (300 W, 4 min, 0.4 mbar). The atmosphere was replaced with O_2_ (0.4 mbar), and a second plasma treatment was applied for 5 min. Again, the atmosphere was replaced by O_2_ for 2 min and finally repressurized by normal air to normal pressure.

#### 2.1.13 Sterility Testing of the Working Space

The working space within the robot platform was tested for sterility. Therefore, sedimentation plates with Blood agar for bacterial and Sabouraud agar for fungal abundance analysis were placed in the robot working cabinet. Contact plates were applied to test microbial contaminations at the robot, arms, and gripper. The samples were analyzed at the Institute for Hygiene and Microbiology at the Julius‐Maximilians‐University Würzburg.

### 2.2 Engineering

#### 2.2.1 Computer‐Aided Design

Computer‐aided design (CAD) was performed and rendered with Solidworks Premium 2017 (Dassault Systèmes, France). For 3D printing, the files were converted into Standard Triangulation Language (STL).

#### 2.2.2 3D Printing

3D printing was performed by converting the STL files into printer‐specific geometry‐code‐like files using the respective software of the individual printers. Two different printing methods were applied. First, Fused Deposition Modeling (FDM) was used to process polylactic acid (Filamentworld, Germany) and Green‐Tec Pro (Extrudr; FD3D, Austria). For the printer Raise3D Pro 2 (RAISE3D Technologies, USA) the slicing software Ideamaker 3.4.2 of the same company was utilized, while Simplify3D 4.1.2 (Simplify Software, USA) was used for the slicing in advance of printing with German RepRap X500 (German RepRap, Germany).

Second, Stereolithography (SLA) was conducted to print peripheral parts and molds with higher surface quality. These processes were performed by the printer Form 2 (Formlabs Inc., USA) using the software Preform 3.0.1 and the materials Grey Resin as well as Model V2 Resin (Formlabs Inc., USA). Stereolithographic‐printed parts were washed in isopropanol for 10 min using the Form Wash device (Formlabs Inc., USA). Afterward, the parts were cured with UV‐light at 60 °C for 30 min using the Form Cure device (Formlabs Inc., USA).

#### 2.2.3 Production of Tailored Silicone Components

PDMS positioning mats were generated as previously published.^[^ ^53^ ^]^ Briefly, the required structure was designed by CAD and 3D‐printed. A negative mold was created by using Dublisil 15 (Dreve Dentamid, Germany), a two‐component silicone. After plasma activation of the mold surface using a plasma cleaner device, the two‐component PDMS (SYLGARD 184; Dow Europe, Germany) was mixed by a ratio of 10:1 and cast into the preactivated silicone mold and stored in an oven (HERATHERM; Thermo Fisher Scientific, USA) at 37 °C overnight. The next day, the PDMS‐molded part was removed from the Dublisil mold and autoclaved once before final use to ensure proper crosslinking.

### 2.3 Software Development

#### 2.3.1 Robotic Manipulator

The dual‐arm robot Motoman CSDA10F (Yaskawa Europe, Germany) was used as a manipulator. Employed by the industrial robot controller FS100 (Yaskawa Europe, Germany) tasks and work sequences were programmed. A library of tasks and working sequences, e.g., pipetting, dispensing, or the transfer of samples was generated and is listed in Table S1 (Supporting Information).

#### 2.3.2 Programmable Logic Controller

The Programmable Logic Controller (PLC) SIMATIC ET 200SP (Siemens, Germany) was used to map all cell culture processes by combining elements from the task and work sequence library of the robot. Furthermore, task parameters such as pipetting volume, incubation time or temperatures were provided in respective variable sets. The entire cell culture process was mapped by combining elements from the task and work sequence library.

#### 2.3.3 Image Acquisition

The microscope with motorized motion (045‐100204; Opto, Germany) was incorporated into the platform. Enabling integrated real‐time image acquisition of the Neubauer chamber. A configuration of LEDs shielded by a light diffuser was set up to emit light into the chamber. The chamber was placed within an adapter that can be moved using the motorized motion to predefined positions for image acquisition. Captured images were stored on a remote computer for subsequent analysis.

#### 2.3.4 Automated Cell Count

Cell count was automated using a detection pipeline based on You Only Look Once (YOLO) open‐source deep learning model used for object detection in images and videos.^[^ ^54^ ^]^ YOLO and comparable deep learning models have been recently utilized for use in biomedical applications ranging from cell classification to medical image analysis.^[^ ^55^, ^56^, ^57^ ^]^

The dataset was annotated and managed using the RoboFlow platform. The pre‐processing includes sliding window crop and gray morphology operations implemented by utilizing Python 3 libraries (Pillow 8.3.2, OpenCV 4.5.3, NumPy 1.21.2 and PyTorch 1.9.0). Python codes for preprocessing, training, and inference in the detection pipeline were executed over Jupyter Notebooks on a local server. For further information on the automated cell count, please find the code and explementary data on our Github repository (https://github.com/Fraunhofer‐ISC/Automated‐Cell‐Count/tree/main).

### 2.4 Statistical Analysis

The data presented in this paper are shown as mean ± standard deviation if not marked differently. The calculation was done either in Excel (Microsoft Corporation, USA) or Prism 9 (GraphPad Software, USA). Statistical analysis was performed using Prism 9.

## 3 Results

### 3.1 Technical Development

**Figure 1:**
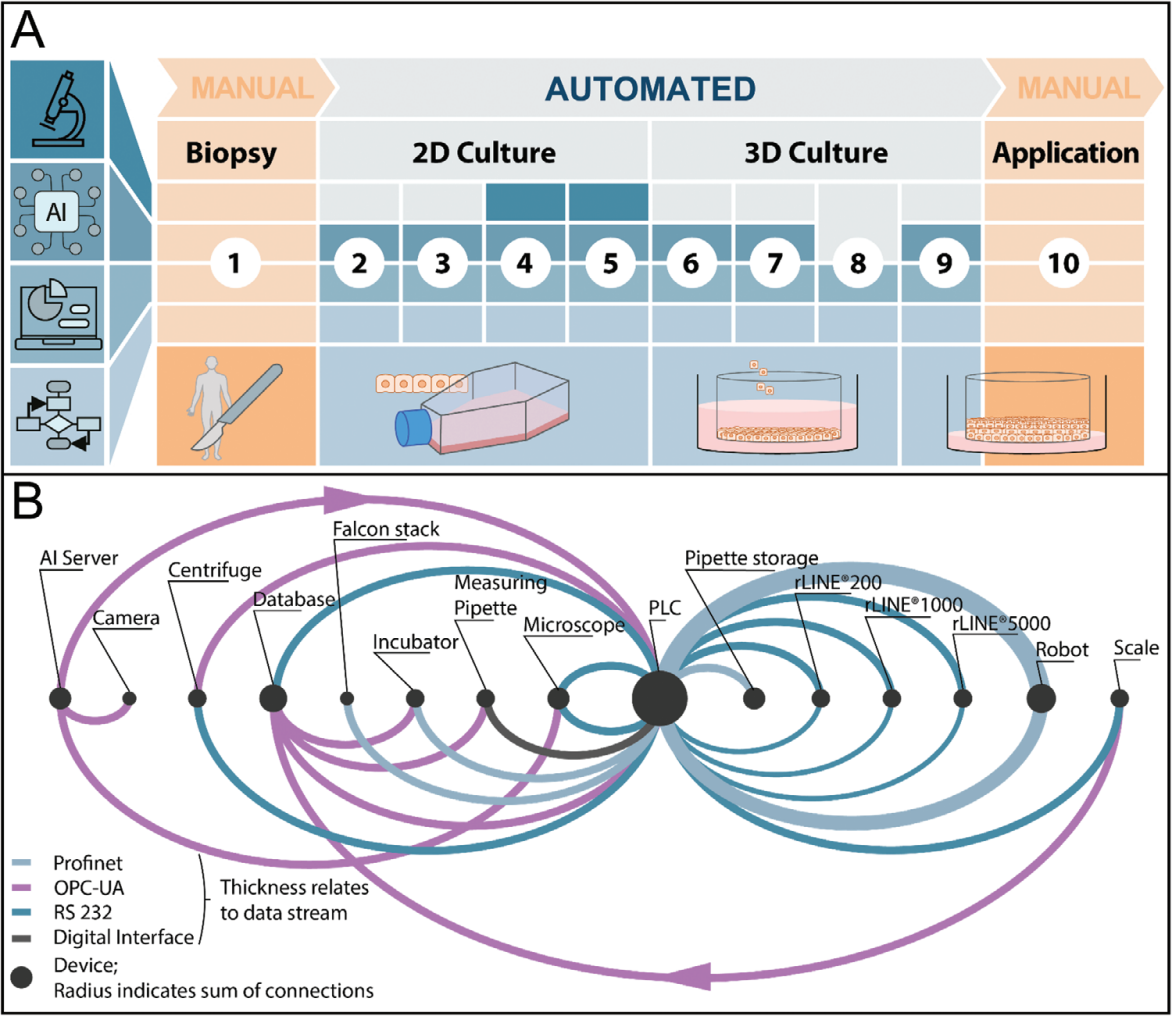
Workflows for automated tissue engineering. A) The icons on the left represent the use of automation technologies, from the top an automated microscope, neural networks for data processing, collection of process data in a cloud, and translation into an automated process using a PLC and step sequences. General workflow derived from manual SOP: (1) Manual cell isolation; (2) 2D cell seeding; (3) medium exchange in T‐flask; (4) cell detachment; (5) cell count and resuspension; (6) 3D cell seeding; (7) add proliferation medium; (8) set to ALI; (9) medium exchange in cell culture plate; (10) manual evaluation and further use of the tissues. B) Illustration of the communication interfaces, data flow is clockwise. The thickness of the lines represent an abstraction of the data flow intensity between the respective partners.

To overcome barriers 1–5, a robotic system was implemented for the automated generation of in vitro tissue models. As a proof of concept, the manual generation of RHE and hATM was translated into automated processes. The workflow for the automated system was derived from the manual SOP and the analysis of videos recorded during the manual process by laboratory staff (Figure S1, Supporting Information). The workflow for tissue model generation was divided into ten individual steps, of which eight core steps were automated (**Figure** **1**A3‐9). Applied automation technologies included the use of the automated microscope, neural networks for data processing, collection of process data in a cloud, and translation into an automated process using a PLC and step sequences. The workflow included further the following procedures. 1) Primary cells were obtained from manual isolation of a biopsy. 2) For cell expansion, cells were seeded into standard cell culture T‐flasks and 3) the culture medium was regularly changed to ensure optimal conditions for cell proliferation. 4) Once the cultures reached ≈80% confluence, the cells were enzymatically detached with mechanical stimulation. 5) After centrifugation and resuspension in a defined volume, automatically acquired images from the automated microscope were analyzed by a neural network developed to count living and dead cells. 6) This analysis allowed the cell concentration to be set in accordance with the SOP. The correct concentrated cell suspension was transferred to cell culture inserts placed on a customized robotic well plate (RWP). 7) After cell attachment, the medium compartment of the cell culture plate was filled with culture medium. 8) The next day, the cells were shifted to ALI, and the medium was changed to allow keratinocyte differentiation. 9) For tissue maturation, the culture medium was regularly changed three times per week. 10) The automatically generated tissue models were then used for further downstream applications. The dual‐arm robot concept with human‐like kinematics allows the use of generic laboratory materials for the manual process, which was further analyzed to identify necessary containers and instruments. Essential consumables (sterile pipettes, centrifuge tubes, culture flasks, and well plates) for tissue model generation have been defined. The communication of the PLC with the robot and peripheral instruments via different protocols and the data volume is displayed in Figure 1B. Further explanation of the structure of robot jobs and communication between PLC and robot can be found in Figure S2 and Table S1 (Supporting Information).

**Figure 2:**
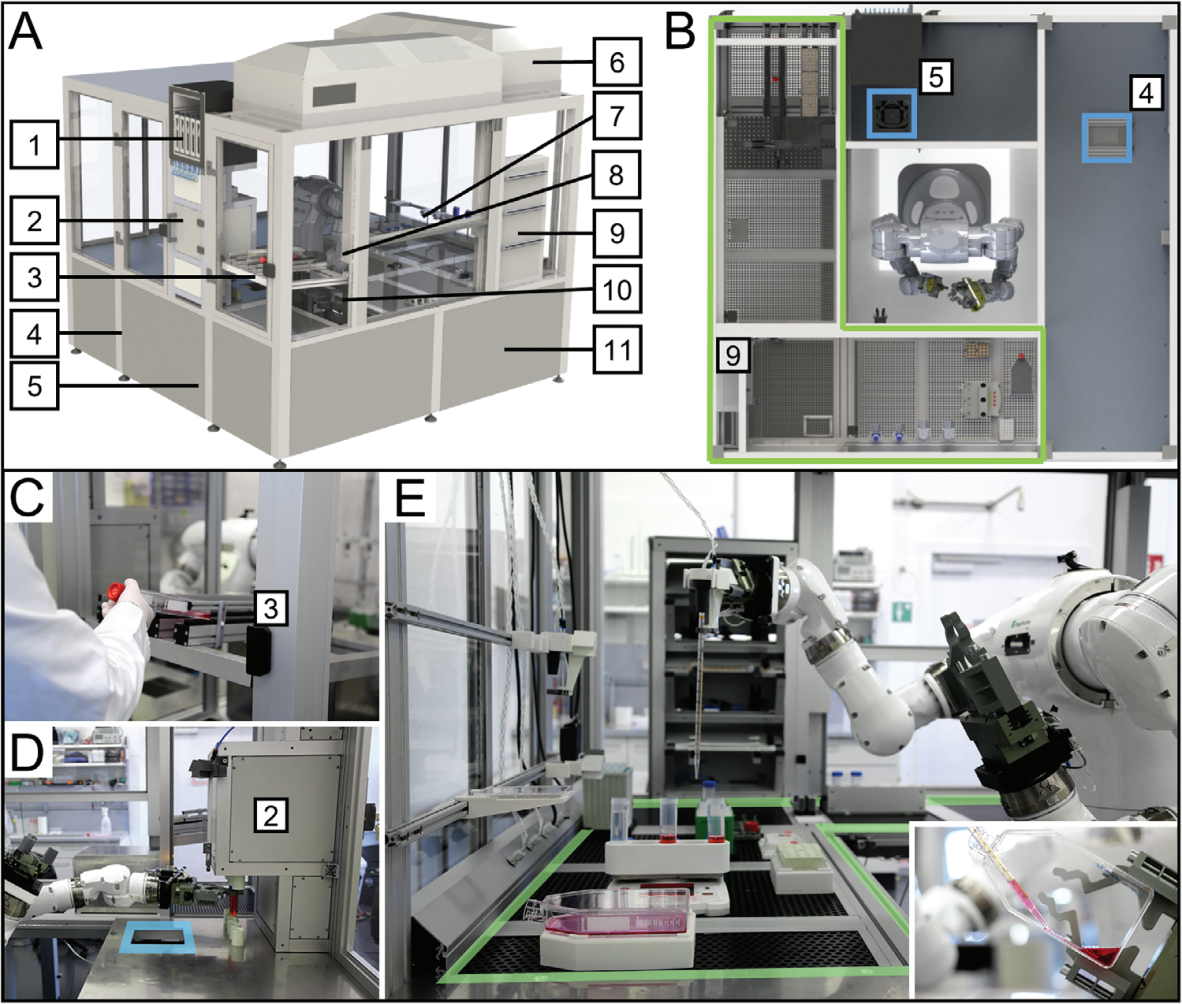
Complete robotic system. A) The working area of the robot is designed to ensure that all tailored devices and standard laboratory equipment can be installed within reach of the robot. (1) falcon stack 4 °C; (2) falcon stack room temperature (RT); (3) material port; (4) incubator; (5) centrifuge; (6) laminar flow; (7) pipette rack; (8) robot; (9) pipette separation system; (10) microscope; (11) controlling unit [PLC]. B) Top view of the whole system; (4), (5) access to centrifuge and incubator; (9) pipette separation system; (green framed area) sterile area secured via laminar flow. C) Technician handing over 2D cell culture in T‐flask to the material port. D) Robot taking released culture medium out of RT stack to transfer it to the sterile area. E) Robot using the high‐volume pipetting device to refill culture medium.

**Figure 3:**
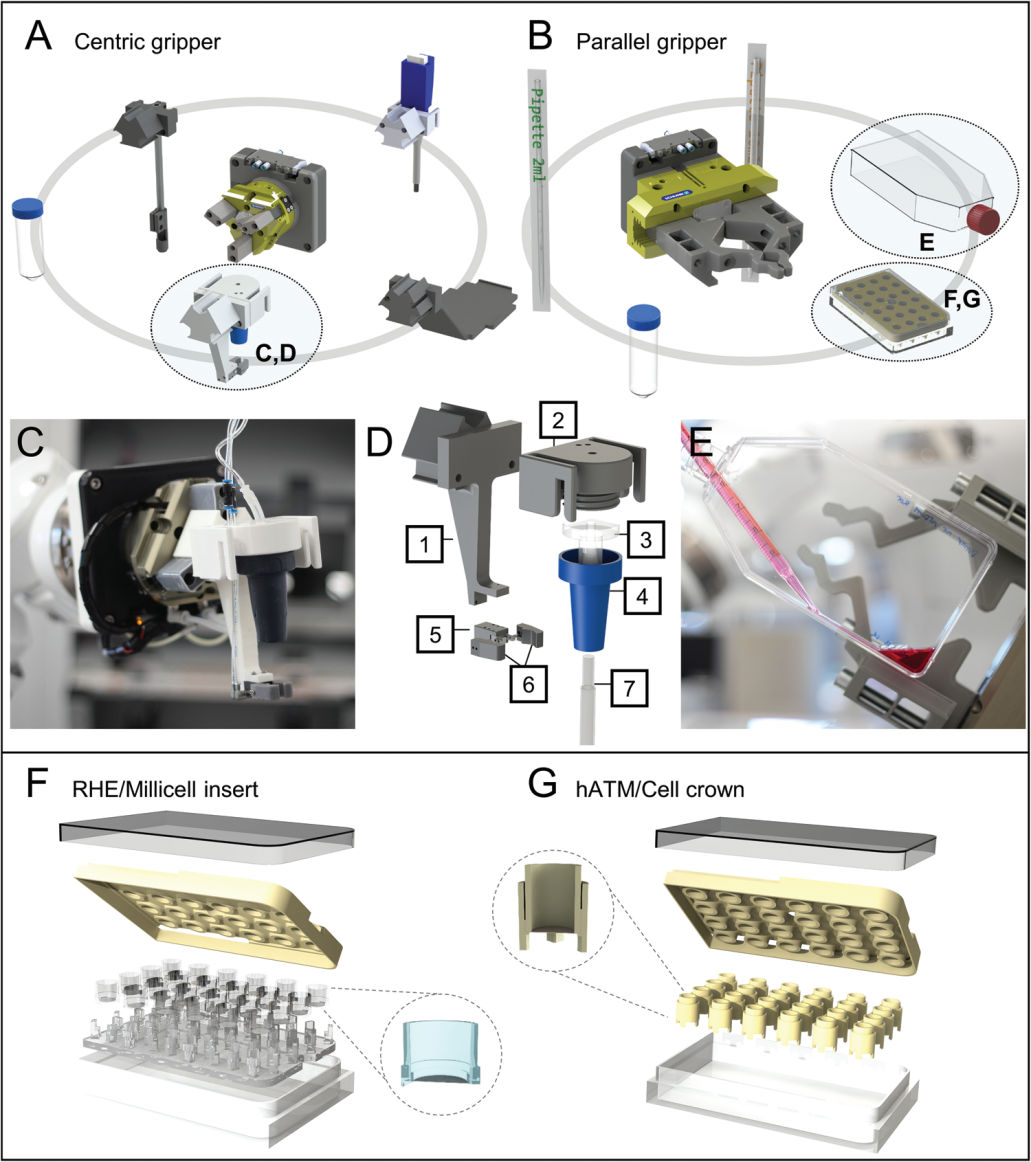
Design of devices. Automated tissue model generation required the adaption of the robotic platform and of the laboratory material. To allow maximum flexibility in handling various geometries of equipment, two gripping mechanisms were implemented: parallel and centric gripping. A) For the mobilization of different tools, as well as the opening of screw caps and the transport of centrifuge tubes on the lid, the choice is made for the self‐centering properties of the centric gripper. B) Parallel gripping fingers can handle T‐flasks, centrifuge tubes on the shaft, microtiter plates, and various measuring pipettes. C) The high‐volume pipette (HVP) is developed based on the standardized hardware interface for the centric gripper. D) It is adaptable to use corrugated tips with a nominal volume from 2 mL up to 50 mL. (1) universal grip adapter with pipette centering; (2) headpiece with universal magazine adapter; (3) silicone attachment; (4) connector hull; (5) pneumatic scissors gripper; (6) pipette centering gripper; (7) disposable graduated pipette. E) The combination of parallel and centric gripper allows complex sample manipulation like the dosing of liquid inside of T175 cell culture flask. To ensure the correct positioning of the cell culture transwell inserts or cell crowns, a plate holder as well as an insert holder were integrated. F) The inner structures of the RWP enable the cultivation of 24 tissue models on a shared medium compartment. The positioning mat and plates are adapted for RHE culture using Millicell inserts, and G) cell crowns with a biological matrix for hATM.

**Figure 4:**
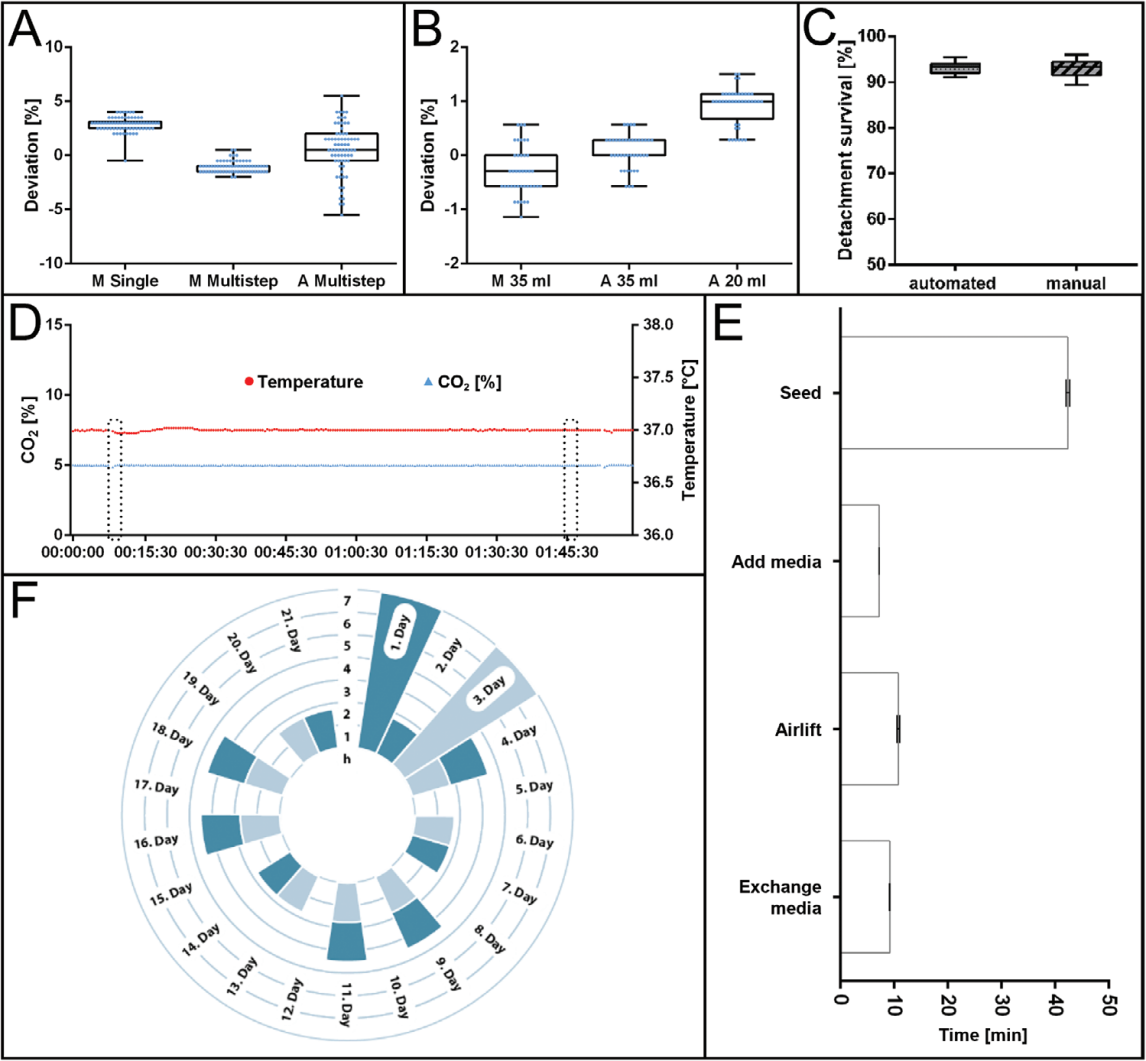
Process monitoring. Comparison of data collected during the automated process with data from manual handling steps. A) Dosing accuracy for seeding the cell suspension *V  =  200 *µL. In comparison rLine(R) 5000 incl. automated tip assembly (*n  =  71*), single‐channel pipette nominal volume 1000 µL (*n  =  72*), Multistepper with 10 mL attachment (*n  =  71*). B) Dosing accuracy at setpoint 35 mL and 20 mL with the robotic system compared to dosing with the measuring pipette (*n  =  40*). C) Survival rate of hEK after accutase‐mediated detachment. Whiskers indicate minimum and maximum values (A–C). D) Exemplary plot of the online‐acquired measurements of CO_2_‐concentration and temperature of a representative 2 h period. The marked areas indicate the unloading and subsequent loading of well plates, showing constant conditions. E) Hands‐on‐time of the robotic system, including the automated preparation of pipettes and transfer of materials into the workspace, loading/unloading of the incubator (*n  =  6*). Graphs show mean values with SD. F) A schedule displays the time occupancy of the plant to produce and culture 14 batches of RHE over 21 days (*14 × 24 RHE  =  336 RHE)* within a timeframe of 21 days and 7 h per day representing a workday of manual operators. Seven batches are produced on d1 (dark blue) and d3 (light blue) respectively. The following occupancies represent the culture steps of Airlift and media exchange.

**Figure 5:**
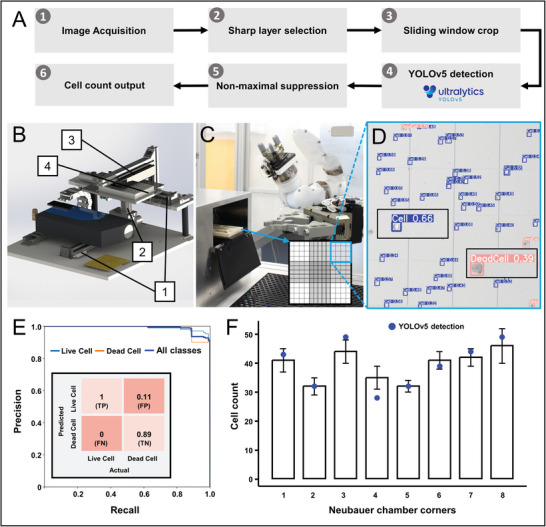
Automated cell classification and counting using a custom trained YOLOv5 based detection pipeline. A) The workflow starts with (1) acquiring multi‐layered images using the microscope; (2) selecting sharp images; (3) employing a sliding window technique to generate cropped images sized to the Neubauer chamber grid; (4) using a YOLOv5‐based detection pipeline to differentiate between live and dead cells; (5) implement non‐maximal suppression to eliminate duplicate detections; (6) finalize the cell count by analyzing labeled instances of live and dead cells. B) The automated microscope comprises (1) motion stages, (2) an objective lens, (3) a light diffuser, and (4) a well plate carrier. C) The Neubauer chamber is positioned within a holder inserted into the microscope loading unit by the robotic parallel gripper. D) Live and dead cells are detected from microscope image acquisition. E) Mean Average Precision (mAP) of 0.98 is achieved for detection of both classes. Moreover, high values of true positive (TP) and true negative (TN) and low values of false positive (FP) and false negative (FN) in the confusion matrix (inset), confirm the efficiency of the classification. F) To validate the automated cell‐counting process, we performed a manual cell‐counting experiment with the collaboration of 15 expert biologists. Graphs show mean values with SD, blue points indicate the prediction of the automated cell count.

**Figure 6:**
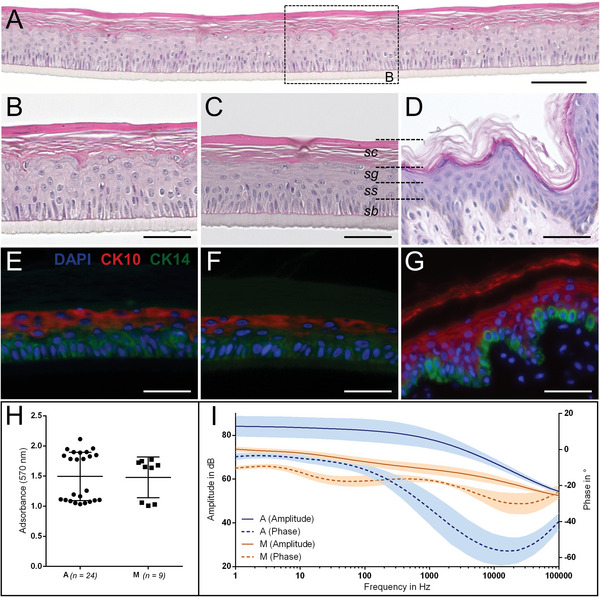
Biological evaluation of automated RHE production. A–D) HE‐stained sections of automated RHE (A,B), manually generated RHE (C), and native skin (D). E–G) Immunofluorescent analysis of biological markers CK10 (red) and CK14 (green). Nuclei are stained blue by DAPI. Scale bars = 100 µm (A), Scale bar = 50 µm (B–G). H) Viability analysis by MTT assay comparing the metabolic activity of automatically produced (A *n  =  24*) and manually generated RHE (M *n = 9*). I) Analysis of barrier function via impedance spectroscopy. The Bode plot for a representative experiment shows the averaged amplitude and phase response for the automatically cultivated RHE compared to the manual controls (A *n  =  12*; M *n  =  6)*. Graphs show mean values with SD, H–I.

The derived automated process is based on the steps of the human experimenter. **Figure** **2**A shows the developed automated system including a variety of components: The whole operation area of the robot is secured against external access by a safety housing. It is divided into two different working environments (Figure 2B): A sterile working area with laminar flow units (Figure 2A‐6,E) and an unsterile bench used for the integration of subsystems (Figure 2C,D). The hygiene concept and the results of a microbiological analysis are presented in Figure S3 (Supporting Information). All components have been arranged in a space‐efficient and process‐dependent manner. Thereby, processes were optimized, and contamination risks were minimized. A cytomat 2 C‐LIN (Thermo Fisher Scientific, USA) is integrated into the benchtop (Figure 2A‐4, B‐4). The robot can load individual well plates independently. For the storage of culture media in 50 mL centrifuge tubes, a storage unit was placed in the plant that provides storage capacities at room temperature and 4 °C (Figure 2A‐1,2). As shown in Figure 2E, the robot discharges a prepared culture medium for exchange of liquids in T‐flasks (insert). The PLC (Figure 2A‐11 placed behind the cover) controls all peripheral devices. Cell cultures, e.g. cell culture plates and T‐flasks are placed into the material port by the laboratory staff (Figure 2A‐3,C), whereas pipettes are stored in the pipette separation system (Figure 2A‐9,E). A demonstration of the robot's interaction with a variety of peripherals and materials can be found in Video S3 (Supporting Information).

To ensure the flexible interaction of the robot with its environment, the mounted grippers must allow the handling of generic materials and laboratory instruments. **Figure** **3**A shows the design of the parallel and Figure 3B of the central gripper as well as respective materials and tools. The grippers are designed in a modular manner in order to facilitate handling of various laboratory consumables (e.g., T‐flasks, centrifugation tubes, etc.) in consideration of their shape, contours, and orientation. The equipped fingers were manufactured by different 3D printing technologies that allowed faster development, adaptations, and iteration cycles. The parallel grippers can handle square geometries, such as T‐flasks, well plates, and the unwrapping of pipettes. Whereas the central gripper opens and closes T‐flasks and centrifugation tubes by screwing the lid. Furthermore, via the standardized hardware interface, the central gripper operates tools, such as the high‐volume pipette (HVP), which can inherit different sizes of serological pipettes (Figure 3C). The HVP was designed specifically for this interface, and the exploded view in Figure 3D shows how the combination of 3D printing to produce the universal grip adapter with pipette centering (1) and the headpiece with universal magazine adapter (2), combined with generic materials such as the silicone attachment (3), connector body (4), and pneumatic scissors gripper (5), allows the development of automated tools based on manual applications. The combination of both grippers and the variety of tools allows complex manipulation of samples. To ensure the orientation of the pipette tip, the angle grippers (6) are designed for the geometry of either 2 mL or 10–50 mL nominal volume pipettes (7). Figure 3E displays how the robot can use the HVP in combination with the parallel gripper for fluid dosing within a T175 cell culture flask.

For the manual RHE process, the inserts were placed individually in six‐well plates. To ensure robust handling of tissue models by the robot, a culture plate for 24 RHE was equipped with a PDMS‐casted positioning mat and the 3D‐printed positioning lid, securely holding the inserts in place for robotic manipulation (Figure 3F). By this procedure the correct position of 24 transwell inserts is secured and supports accurate handling. The RWP has a shared culture medium compartment which allows the one‐step exchange using the HVP (Figure 3B,E). For the culture of hATM, minor changes in the culture plate allowed the use of cell crowns containing the porcine scaffolds as shown in Figure 3G.

To ensure process accuracy and allow troubleshooting, crucial process parameters (dosing accuracy, cell viability after detachment, incubation parameters, and process times) were monitored and analyzed, as shown in **Figure** **4**. The accuracy of the robotic platform to transfer fluids was evaluated in comparison to manual operators by weighing dosed volumes of water. The accuracy of the HVP to transfer defined volumes (35, 20 mL) was examined (Figure 4A). For the target volume of 35 mL, the HVP achieved an accuracy of *35.03 ± 0.109 g*, compared to *34.90 ± 0.037 g* for the manual operator. For the target volume of 20 mL, the HVP achieved an accuracy of *20.17 ± 0.110 g*, compared to *19.95 ± 0.140 g* for the manual operator. In addition, the ability to accurately transfer small volumes (5–5000 µL) was addressed including the Sartorius rLine series. For cell seeding the Sartorius rLine 5000 is used in the multistep pipetting approach (Figure 4B). The analysis revealed an accuracy of *200.7 ± 6.012 mg* of the set seeding volume of 200 µL by the Sartorius rLine 5000 (Figure 4B). The manual operator using a single channel dispense pipette showed *198.1 ± 1.832 mg* (M Single) and using the Multistepper seems to be most accurate with *198.9 ± 1.304 mg* (M Multistep). For efficient cell harvest, the enzyme‐mediated cell detachment requires exact incubation timing and gentle handling to allow sufficient cell retrieval and survival.^[^ ^58^ ^]^ A special device, for standardized detachment of 2D adherent cell cultures, was developed (Figure S4, Supporting Information). It facilitated a high cell survival of ≈95%, comparable to the manual cell detachment (Figure 4C).

In general, the reproducibility of in vitro experiments is strongly dependent on incubation conditions, such as constant temperature and CO_2_. Therefore, the incubation parameters of the incubator were detected. Both parameters (temperature of 37 °C and CO_2_ concentration of 5%) were stable over a time period of 2 h (Figure 4D). The marked areas indicate the unloading and subsequent loading of well plates, showing constant conditions. If a drift in temperature or CO_2_ concentration persists, the system can alarm the user.

The processing time of biological material—externally of the cell incubator—is important to consider during automated processes, as the material is exposed to changing environmental factors (temperature, sterility, etc.), and for production prediction. The time required for the automated seeding procedure *T_Seeding_  =  42.4 min*, addition of differentiation media *T_Add_  =  7.2 min*, the introduction of the ALI *T_ALI_  =  10.8 min*, and the medium exchange *T_Exchange_  =  9.24 min* (Figure 4E). Based on these process times, a schedule for the production of 336 RHE (14 batches of 24 tissue models) is illustrated in Figure 4F.

**Figure 7:**
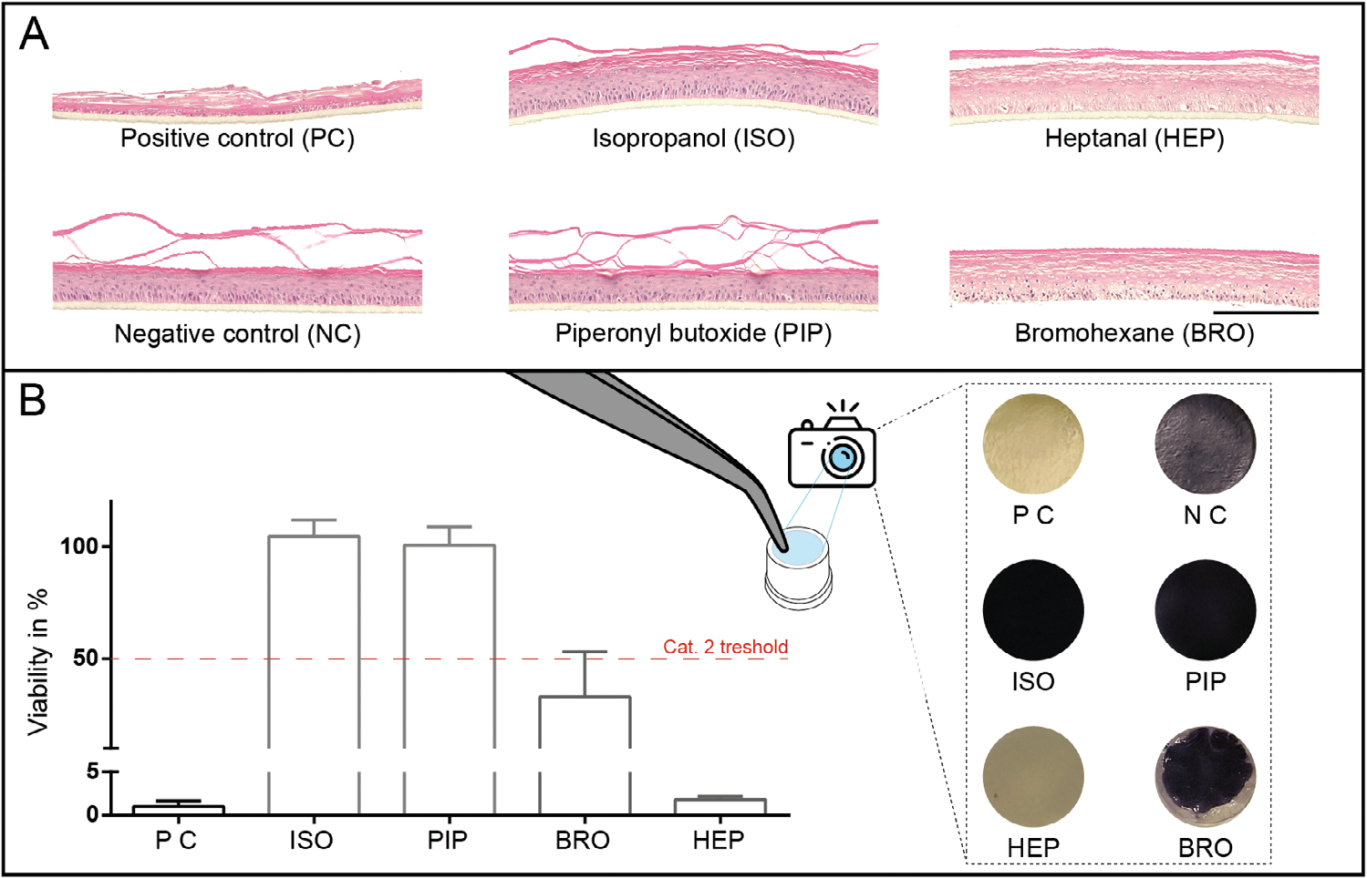
Skin irritation test adapted by OECD 439. A) HE stained sections of automatically generated RHE, observed 42 h after irritation. Scale bar = 200 µm. B) Quantitative MTT normalized on NC; pictures of RHE after MTT incubation (*n = 6*; three independent experiments). Graphs show mean values with SD, B.

**Figure 8:**
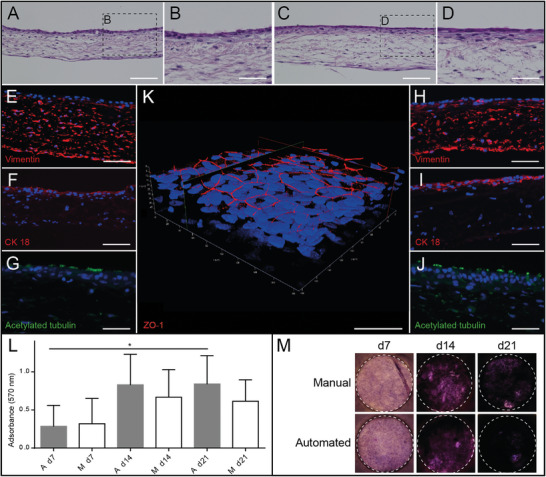
Comparative analysis of manually and automatically produced hATM. A–D) Histological analyses revealed no obvious morphological differences comparing automatically (A,B) and manually (C,D) produced tissue models. E–J) Immunofluorescent staining confirms cilia‐like structures and differentiated epithelial cells in tissue models, with consistent morphology across E–G) automatically and H–J) manually produced samples, and cell nuclei are stained with DAPI. K. 3D reconstruction of hATM with apical tight junction marker ZO‐1 reveals functional tissue polarization. L–M) Quantitative (L) and qualitative (M) MTT data showed significantly higher cell metabolism in automatically produced tissue models comparing d7 and d21. Graph shows mean values with SD, L (*n =  3*). *:  *p*‐value of unpaired *t*‐test with Welch's correction < 0.05. Scale bar = 100 µm (A,C,E,F,H,I); scale bar = 50 µm (B,D,G,J,K).

The generation of reproducible tissue models requires an accurate number of cells for the initial seeding process. Therefore, an automated cell counting procedure was implemented in the robotic platform. Its pipeline includes image acquisition, identification of the correct focus level, a sliding crop window selection, detection by YOLOv5 for cell identification, non‐maximal suppression, and output of the cell number (**Figure** **5**A). In detail, images of cell‐loaded Neubauer chambers were acquired by the in‐built automated microscope (Figures 2A‐10, and 5B,C). Subsequently, the detection pipeline based on the open‐source machine vision model YOLOv5, effectively identifies, and quantifies live and dead cells (Figure 5D). The training dataset consisted of 77 images acquired via four Neubauer chamber image acquisition with the microscope. In total, it includes 7142 instances of cells (5966 live cells and 1176 dead cells). Mean Averaged Precision (mAP) is calculated as 0.986 for both classes on a test set of eight images. Moreover, a confusion matrix was generated to assess the performance of the model. The high values of true positive (TP) and true negatives (TN) validate the efficiency of the classification. Furthermore, the absence of false negatives indicates the detection and classification of all live cells resulting in a recall score of 1. However, the presence of false positives suggests that ≈5.5% of dead cells were misclassified. Nevertheless, the model maintains a relatively high accuracy of 94.5% in detection of dead cells (Figure 5E). For further confirmation of the efficiency of the model, we conducted an experimental validation with the collaboration of 15 biologists with experience in cell counting. Our test dataset comprised eight images of Neubauer chamber corners (grid with corners depicted in Figure 5C,D). Each participant counted the number of live and dead cells. Subsequently, we applied our YOLOv5‐based algorithm to the same set of images. The aim of this validation experiment was to use the manual cell counts as a reference point and determine the extent to which the counts from our automated detection pipeline aligned. The relative mean error between manual and automated count is calculated as 6.5% (Figure 5F).

### 3.2 RHE Analysis

The automatically generated RHE were analyzed regarding their morphology, the appearance of tissue‐specific marker proteins, cell viability, and reproducibility. The histological analysis showed that RHE had a consistent quality over the whole surface of the cell culture membrane, as demonstrated in **Figure** **6**A,B. Furthermore, the microscopic analysis of the HE staining confirmed that the RHE has the same morphological features compared to the manual control (Figure 6C). Both contain the epidermis‐specific formation of keratinocytes proving the differentiation and maturation of hEK toward a functional epithelium in vitro similar to native human skin (Figure 6D). The cells indicate proliferation from the stratum basale (sb) and migration upward, thereby flattening as shown by hEK in the stratum spinosum (ss). The cells further accumulated keratin granules within the stratum granulosum (sg) before the hEK entered the stratum corneum (sc). Immunohistological analysis of cytokeratin (CK) 14 and CK 10 in automated RHE, manual RHE, and native skin shows similar localization of marker proteins (Figure 6E–G). CK 14 is found in cells of the sb, whereas the epidermis‐specific differentiation marker CK 10 is localized in differentiating cells of the ss and sg. The viability of RHE was evaluated after 19 days of culture using an MTT assay. The measurement of the optical absorbance at 570 nm revealed comparable viability of cells between automated and manual models (Figure 6H). On day 19, automated models are fully developed, recognizable by the amplitude and phase response with the corner frequency in the range of 1000 Hz (Figure 6I).^[^ ^51^ ^]^

The differentiated models were subjected to manual irritation testing on day 19 in alignment with OECD test guideline 439 to assess if the automatically generated models are applicable for regulatory accepted testing. Two classified substances from each of the groups “No Category” and “Category 2” were tested. The histological analysis of RHE for all test groups (**Figure** **7**A) and MTT assay allowed a qualitative and quantitative analysis of the skin irritation after 42 h (Figure 7B).

The positive control fulfills the criterion of viability of less than 10% and thus demonstrates the suitability of the test system. Category 2 substances (1‐bromohexane and heptanal) were clearly categorized by a viability of less than 50% compared to the negative control. As expected, the two substances of “No category” (isopropanol, piperonyl butoxide) did not reduce viability (Figure 7B).

### 3.3 Human Airway Tissue Model Analysis

In a parallel workflow, we generated hATM manually and automatically using cells from the same donors. In the automated process, the robot performed cell seeding, medium exchange, and introduction of the ALI. Subsequently, the tissue model morphology was comparatively assessed using light and immunofluorescent microscopy. HE stained samples showed that automatically (**Figure** **8**A,B) and manually (Figure 8C,D) produced tissue models that built a continuous epithelial layer on the SIS. On the epithelial surface, we observed cilia‐like structures (Figure 8B,D), which were verified in immunofluorescent staining against acetylated tubulin (Figure 8G,J). Furthermore, automatically and manually produced tissue models revealed vimentin‐positive hAF inside the SIS (Figure 8E,H) and CK 18‐positive differentiated epithelial cells (Figure 8F,I). The overall morphology of automatically and manually produced hATM was similar and no significant differences were observed. Additionally, no noticeable variation comparing independent experiments was found. Manually generated hATM are known to be well‐differentiated and were characterized previously.^[^ ^8^ ^]^ For example, these models feature cell–cell contacts, such as tight junctions as indicated by ZO‐1 staining (Figure 8K). Video S4 (Supporting Information) shows that ZO‐1 staining is restricted to the very apical compartment of the epithelial layer, where the tight junctions are localized both in vivo and in vitro.

To analyze cell viability, qualitative and quantitative MTT assays were performed on d7, d14 and d21. Quantitative data indicated similar absorbance at 570 nm comparing samples of manually and automatically produced hATM. In automatically produced hATM, we found a significant increase in absorbance comparing d7 to d21 (*p* = 0.04, Figure 8L). Qualitative images showed a slightly darker coloration of automatically produced hATM at all three time points (Figure 8M).

## 4 Discussion

This study demonstrates the ability of laboratory automation in life sciences to overcome barriers of 3D in vitro models as an alternative to animal testing. In vitro tissue models have become increasingly interesting for regulatory testing prior to clinical trials. The FDA has recently removed the need for mandatory animal tests in the approval process for new drugs.^[^ ^59^ ^]^ In the EU, current intentions in the Parliament of the European Union aim to accelerate the transition from animal to alternative testing for research applications.^[^ ^60^ ^]^ The topic is also moving to the center of public attention, as European citizens' groups have formed against the use of animals.^[^ ^61^ ^]^ Despite the advantages of animal models for past and current research, in vitro test systems, especially 3D cultures, combined with artificial intelligence are likely to reduce and partially replace the need for animal testing in the future.^[^ ^62^ ^]^

To get over the barriers to a routine application of in vitro test systems, a fully automated process in a centralized system concept^[^ ^63^ ^]^ was developed. This project—ReBiA—overcomes the limitations of currently published and commercially available systems.^[^ ^31^, ^32^, ^33^, ^34^, ^35^, ^36^, ^37^, ^38^, ^39^ ^]^ We exploited the approach through the automation of the entire production process of in vitro test systems (Figure 1A), including 2D cell culture, generation and maintenance of 3D tissue models, and the option to perform test assays. Standardized adapters for the centric gripper allowed the implementation of generic tools (Figure 3), and 3D printing facilitated rapid and efficient integration of tools into the robot's workspace. These tools were based on standard equipment used in the manual process such as pipettes for sterile liquid handling. All devices with a communication interface have been integrated into one network, enabling automated, centralized processing and storage of data such as incubation parameters, process times, and dosed quantities (Figures 1B,4). This automated documentation allows seamless tracking of process steps and simplifies troubleshooting to enhance process robustness and standardization. The applied design criteria enabled a flexible, yet robust, platform solution that can be adapted to a variety of different manual processes.

The feasibility of the proposed concept was demonstrated by the automation of the entire tissue engineering process for the generation of RHE (Figure 1A). The human epidermis is an important barrier tissue protecting the human body against external harm and has already been applied for regulatory‐accepted testing.^[^ ^16^ ^]^ Native epidermis‐specific tissue morphology (Figure 6D), generating the main barrier function, was reconstituted with constant quality in the RHE models of either automated (Figure 6A,B) or manual production (Figure 6C), e.g., physiological reconstruction of the epithelium was confirmed by viability analysis (Figure 6H), keratinocyte differentiation, and epidermal stratification (Figure 6E–G). The quality and properties of automatically produced RHE correlate to the manual process. The observed heterogeneity between individual experiments resulted from the use of different donors, which reflects a typical biological variety.^[^ ^64^ ^]^ A difference was found in the epidermal barrier function. RHE of the automated process showed a characteristic drop in amplitude with a corner frequency ≈1000 Hz,^[^ ^51^ ^]^ whereas for manually generated RHE—using cells of the same donor—a second corner frequency between 10 Hz and 100 Hz (Figure 6I) was detected. This indicates a pre‐maturated state^[^ ^51^ ^]^ and supports the hypothesis that automated culture in the RWP improves the development of tissue models. Accumulation of secreted soluble factors within the shared (Figure 3F,G) compared to separated medium reservoirs of manual culture could explain the observation, as a similar effect was observed for hATM (Figure 8).

The data reveals that the automated culture of 3D tissue models is advantageous for physiological tissue formation and maturation. ReBiA also reduces the effects of human handling. Individual steps of the process show similar and higher levels of reproducibility, such as the accuracy of liquid dosing (Figure 4A,B), material handling (Figure 3), and cell counting (Figure 5). A key factor was the integration of feedback loops for critical process steps, e.g. automated cell counting. Therefore, imaging and counting of detached cells using the automated microscope was supported by AI‐based algorithms (Figure 5). Additionally, the automated system strictly follows a preset schedule that ensures consistent incubation times and precisely triggered medium changes. The precise control and implementation of the process facilitated the application of RHE for an irritation test (Figure 7) that acknowledged a suitable level of **reproducibility (Barrier 2),** a prerequisite for successful validation studies according to the OECD guideline 439. As a consequence, the automated production of 3D in vitro test systems will lead to standardization between laboratories in interlaboratory validation studies and thus will support **regulatory acceptance (Barrier 1)**.

Despite the required level of standardization, the automation concept should still allow transferability to different tissue models, generation procedures, and testing methods to comply with the rapidly evolving field of in vitro testing **(Barrier 5 – Flexibility)**. Figure 3F,G illustrates the modification of the RWP to allow the use of different transmembrane systems of synthetic and biological origin. In combination with a modified process sequence, cells of different tissues, and adapted materials, ReBiA facilitates the automated generation of various established transwell‐based barrier tissue models, e.g. intestinal epithelium, blood–brain barrier, or corneal epithelium.^[^ ^10^, ^65^, ^66^ ^]^ This was demonstrated by the swift implementation of the process to generate SIS‐based hATM. Furthermore, the adaptation of the robotic platform for the generation of hydrogel‐based intestinal organoid cultures revealed a superior accuracy compared to manual handling (Figure S5, Supporting Information).

These results highlight the performance of automated systems for repetitive and time‐consuming tasks with consistent accuracy and speed, thereby reducing the risk of human error.^[^ ^67^, ^68^ ^]^ ReBiA ensures the replication of procedures and conditions, resulting in the consistent quality of human tissue models. This advantage of laboratory automation is essential in research and drug development, where standardized and reproducible results are critical and relevant for costs.^[^ ^69^ ^]^ An additional factor for operational costs in life sciences is labor costs. A release of capacities facilitates the optimization of resources such as time, materials, and personnel.^[^ ^68^, ^70^ ^]^ An automated system based on a similar concept proved to reduce personnel costs by more than 75 % in a life science process.^[^ ^71^ ^]^ From our experience in RHE manufacturing, we know that labor costs account for 50% of the manufacturing process, thus allowing a significant reduced pricing of skin models. This effect can further be strengthened by higher production capacities. The impact on production **cost (Barrier 3)** will amplify with increasing production volume.

A comparison of the productivity of the ReBiA system to laboratory staff is not possible, as the automated procedure is not faster due to the required incubation steps that govern the duration of the processes. However, the ability of automation to work continuously elevates its productivity. Figure 4F shows the schedule for the automated production of 14 RHE batches. Notably, only a low amount of production capacity is occupied, which highlights the potential of the system's throughput. It demonstrates high production capacity at full utilization. Assuming 80% utilization, based on measured processing times, the system can start an average of 17 new batches per day while maintaining existing cultures. This results in the creation of up to 510 batches per month, which is equivalent to 12240 RHE, and thus highlights the scalability of the automated process. In combination with reduced barriers for reproducibility, flexibility, cost, and regulatory acceptance, this 24/7 manufacturing capacity significantly supports the **availability** (**Barrier 4**) of tissue models of reproducible quality for the reduction, replacement, and refinement of animal testing.

In summary, the integration of complex centralized automation into biolabs to produce 3D in vitro tissue models entails benefits and challenges. To exploit the full potential, a comprehensive approach is required, including the design of adaptable workflows and materials. Potential drawbacks, such as an increased risk of contamination for a shared media compartment, are mitigated by an improved sterility concept. While automation is suitable for routine tasks, it can limit opportunities for human creativity and innovation. Manual interaction with experiments can lead to unexpected discoveries that may be lost in automated processing.^[^ ^72^ ^]^ This contrasts with the high degree of reproducibility that these sensitive processes require. The optimal approach considers the synergy between automation and human expertise to drive scientific progress while ensuring the well‐being of workers and the integrity of research results.^[^ ^73^, ^74^ ^]^ The interactive user interface and flexibility of ReBiA help to engage staff in the process and enable collaborative development, monitoring, and implementation of processes while achieving a high level of standardization.

## 5 Outlook

Future developments of the ReBiA platform will include the integration of additional technologies to advance the automation of tissue engineering. On the software level, modular programming interfaces will allow the simple implementation of new protocols. The use of AI methods can optimize experimental design, data analysis, and decision‐making capabilities, thereby increasing process efficiency and robustness in a fully automated test pipeline. In addition to production, test and analysis procedures will be implemented. In this way, the system could serve as a platform for combining the generation of in vitro test systems and in vitro testing.

Hardware updates will further enhance robustness through process monitoring using additional sensors and advanced technologies such as machine vision for AI‐guided monitoring of critical process steps, including machine vision‐based control of unwrapped pipettes and the position of individual tissue models on a well plate (Figure S6 and Video S5, Supporting Information). As a consequence, the adaptability of ReBiA will allow the automated generation of more sophisticated and complex biological test systems, e.g. spheroid and organoid‐based models or tissues applied in multiphysiological systems, such as organ‐on‐a‐chip. As these types of 3D in vitro tissue models increase the complexity of possible studies, this integration is expected to provide valuable insights for disease modeling, drug testing, and personalized medicine. Moreover, GMP‐compliant automated systems are envisioned. For these advanced applications, ReBiA could provide a significant improvement of reproducibility, standardization, production capacity and costs.

Based on the presented results, we envision making the system available to the research community to benefit from standardized results. Within the system constraints, the customer could drag and drop their process, send the required materials, and receive the specific in vitro test systems or analytical results in return.

## Conflict of Interest

The authors declare no conflict of interest.

## Ethics Approval Statement

Approval was obtained from the local ethics committee. (see Section 2.1.1)

## Patient Consent Statement

Cell isolation was carried out with the written consent of the donor or the donors' legal guardians. (see Section 2.1.1)

## Supporting information

Supporting Information

Supplemental Table 1

Supplemental Video 1

Supplemental Video 2

Supplemental Video 3

Supplemental Video 4

Supplemental Video 5

## Data Availability

Supporting Information is available from the Wiley Online Library or from the author. The developed machine learning algorithms are stored on a GitHub repository (https://github.com/Fraunhofer‐ISC/Automated‐Cell‐Count). The PLC code (TIA portal project) and raw data are available upon request, please contact the author.
