# Supplementary material for: ReBiA—Robotic Enabled Biological Automation: 3D Epithelial Tissue Production
Source: Adv Sci (Weinh). 2024 Sep 26;11(45):2406608. doi: 10.1002/advs.202406608 (PMC11615785; doi:10.1002/advs.202406608)
Supplement: Supplementary file 1 — Supporting Information [file ADVS-11-2406608-s004.docx]

**ReBiA – Robotic Enabled Biological Automation: Three-Dimensional Epithelial Tissue Production**

*Lukas Königer, Christoph Malkmus, Dalia Mahdy, Thomas Däullary, Susanna Götz, Thomas Schwarz, Marius Gensler, Niklas Pallmann, Danjouma Cheufou, Andreas Rosenwald, Marc Möllmann, Dieter Groneberg, Christina Popp, Florian Groeber-Becker, Maria Steinke, and Jan Hansmann^*^*

# **Supporting Information**

**Abstracted analysis of manual processes**


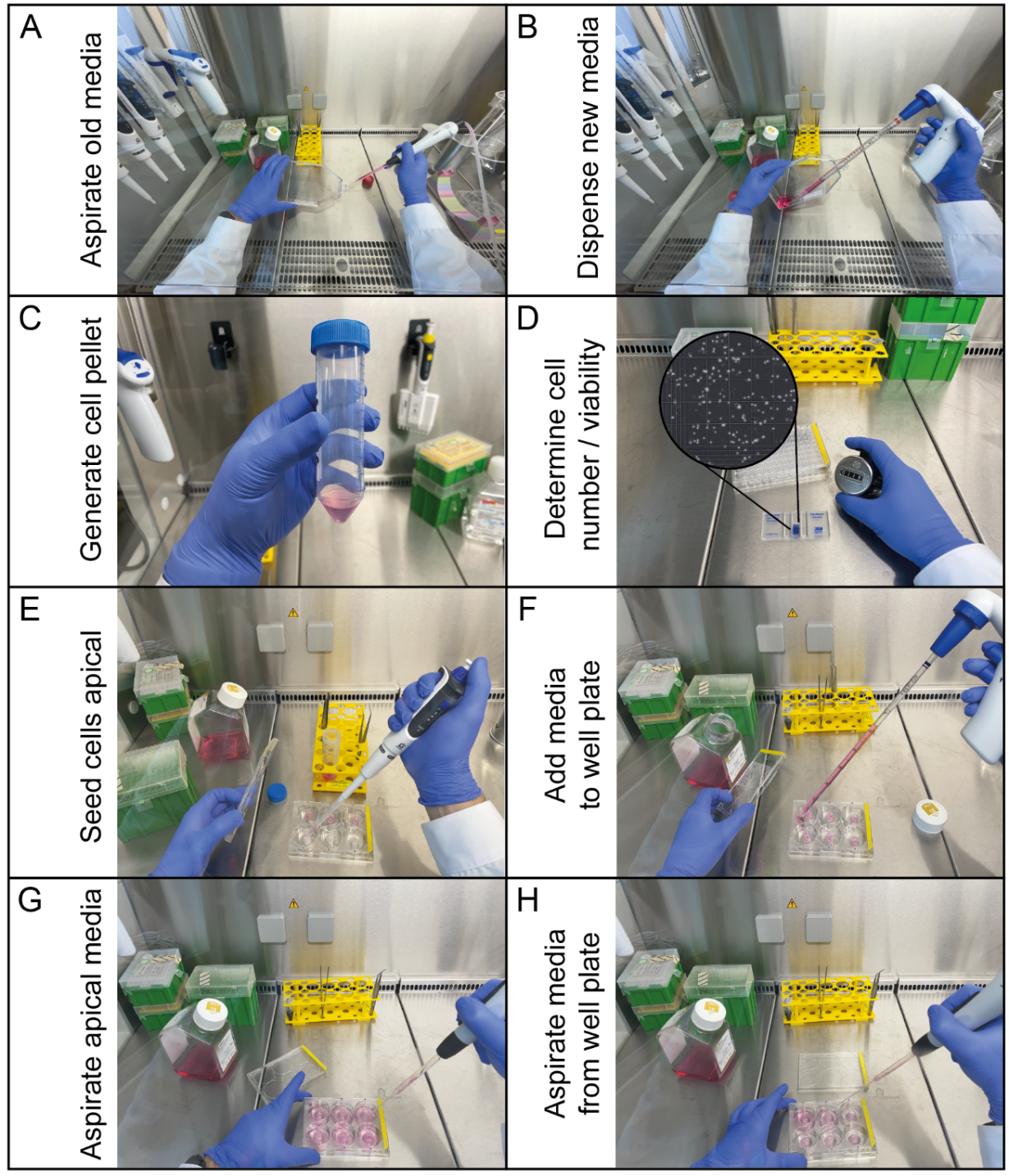


Supplementary Figure 1. Abstracted analysis of the manual process steps for the construction of transwell-and SIS-based in vitro tissue models.

The input to the process is adherent cell cultures in T-flask. In addition to opening and closing, the handling of T-bottles mainly involves the **A.** draining and **B.** filling of various liquids. **C.** After successful detachment from the 2D culture, it is necessary to form a cell pellet. This involves opening, filling and closing centrifuge tubes and integrating a centrifuge. **D.** The number of cells in the pellet formed in this way is determined by counting using a Neubauer chamber. **E.** The concentration of cells can then be adjusted, and the solution applied to the surface of the culture membranes. **F.** In addition to the apical application of fluids, it is also necessary to add fluid to the culture medium compartment. **G.** For the preparation of the airlift culture, it is necessary to allow the aspiration of the medium apically, **H.** while the basolateral side must also be reliably aspirated for regular media changes.

**Orchestration of robot jobs**


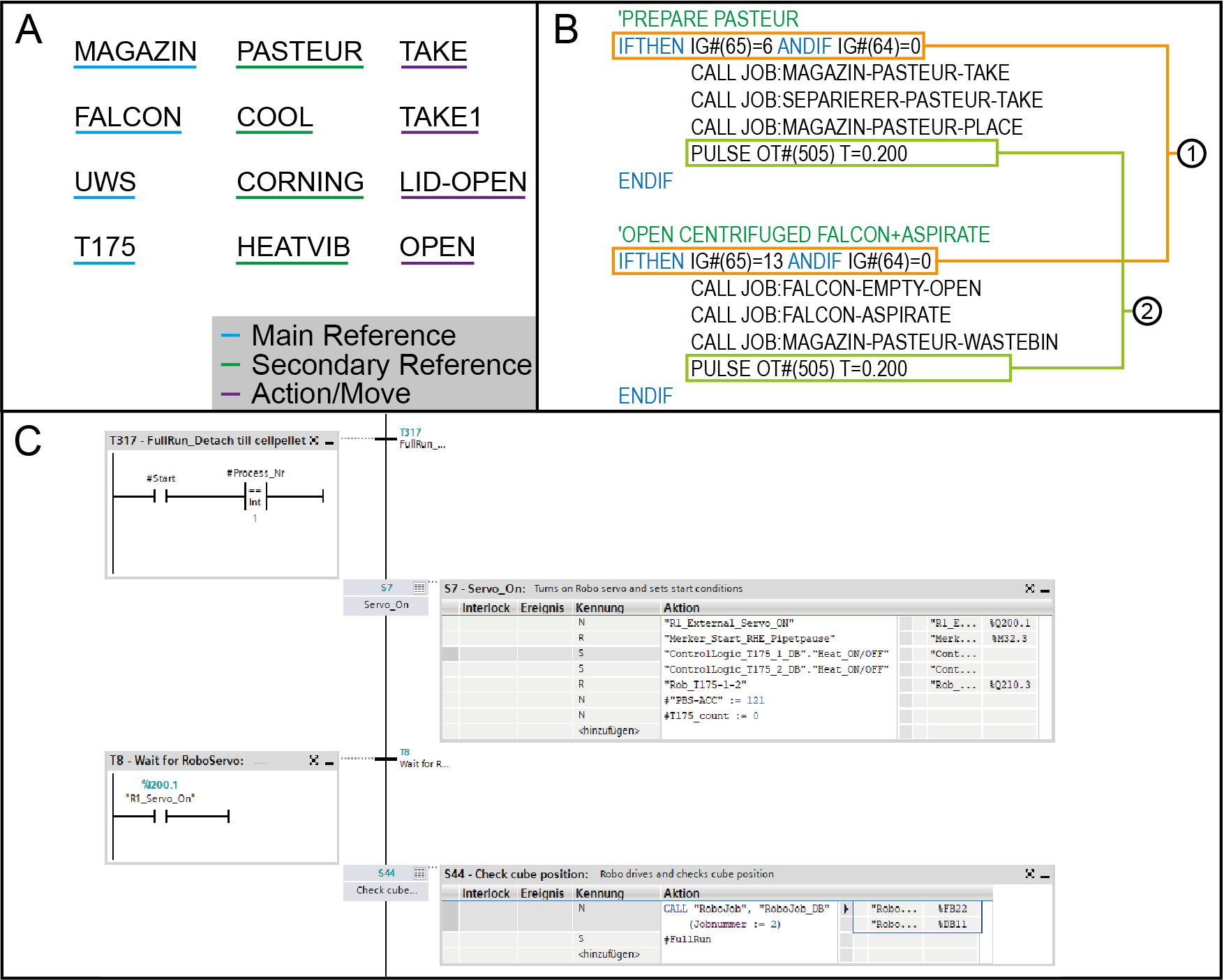


**Supplementary Figure 2. Robot job management.** The nomenclature for robot jobs shown in **A.** was defined to give the programmer a quick overview of the respective function of the job. The names of the jobs are structured according to the following scheme: Reference to the first system that is required, then reference to the material or the target object of the job and finally the action that the robot performs. This catalog of jobs is used by the developer to create call groups that map specific processes. Two examples are shown in **B.** Number one (1) highlights the condition of the call structure, the tags IG#65 and IG#64 read constantly the transmitted value by the PLC. As soon as a number is sent, all listed jobs in the referenced group are processed sequentially. Finally, the robot sends a pulse to output OT#(505), which serves as a transition for the PLC to continue the process (2). **C.** The automated processes, derived by the manual SOP, are efficiently mapped in TIA-Portal via the graphical programming language (GRAPH) as step sequence. Combined robot jobs are called up by the PLC via the Profinet interface with the assigned number. If a new call group is to be created, the jobs available in the job library (Supplementary Table 1) can be recombined and assigned to a number.

**Hygienic testing**


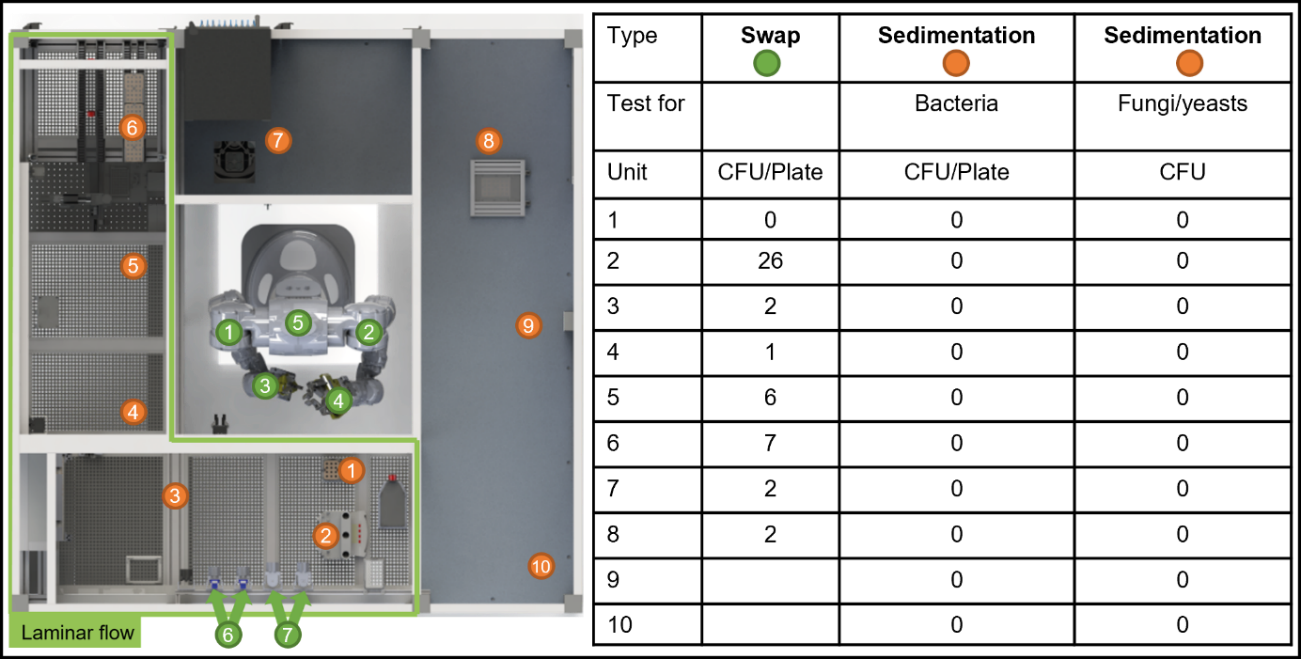
**Supplementary Figure 3.** Hygienic test. A combined test was carried out to determine the cleanliness of the system. The surfaces of the robot and pipettes were tested for bacteria using swab samples, test positions are marked in green. The entire work area was tested for bacterial contamination, yeast, and fungal spores by placing sedimentation plates, these positions are marked in orange.

**Automated detachment of 2D cell cultures**
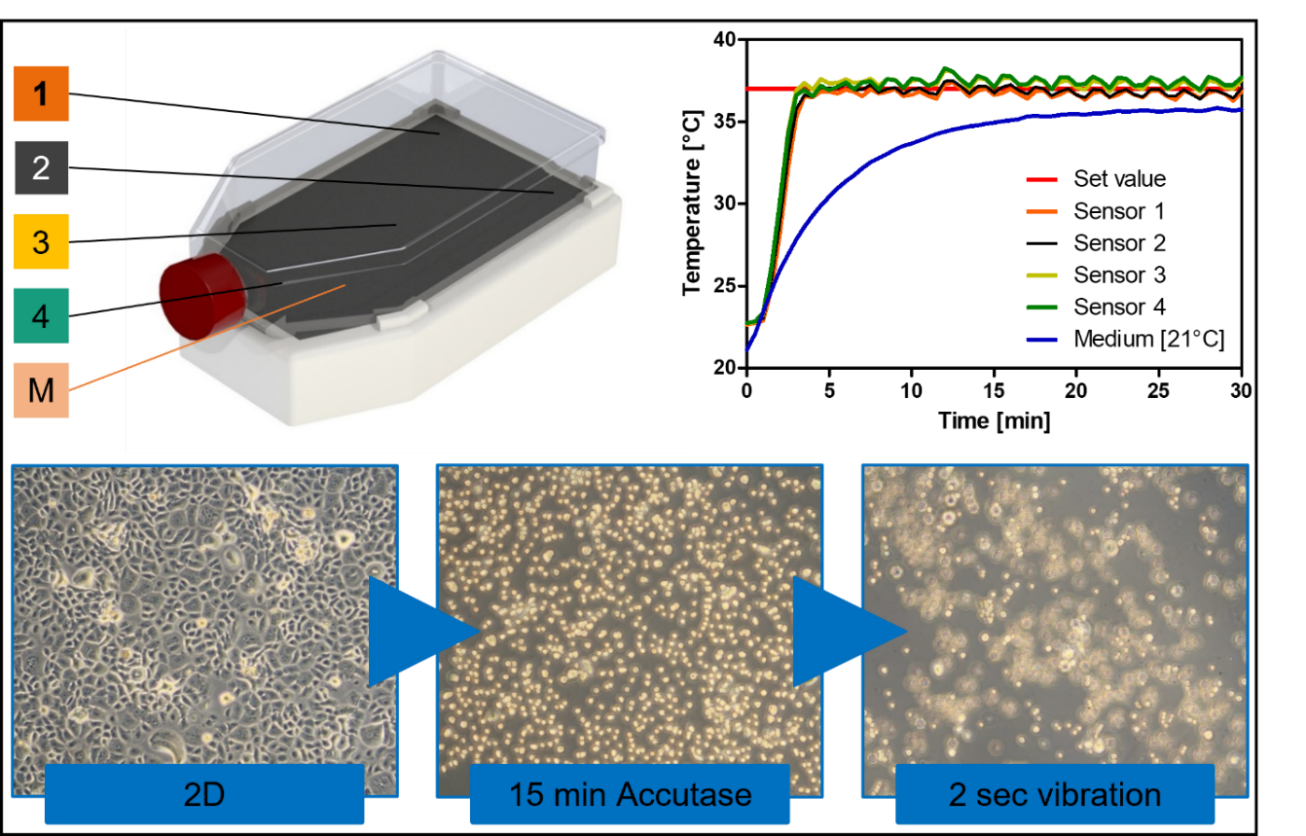


Supplementary Figure 4. Automated detachment of 2D cell cultures

The enzymatic detachment of adherent 2D cell cultures is one key process to generate 3D in vitro tissues. A heating plate was developed to enable an automated detachment process. The even distribution of the temperature inside the T-flask was proven via four individual temperature sensors (1-4) attached to the surface and one sensor inside the liquid (M). By mounting a vibration motor, a defined mechanical stimulus is generated which detaches attached cells from the surface at the end of the incubation time.

**Hydrogel handling**
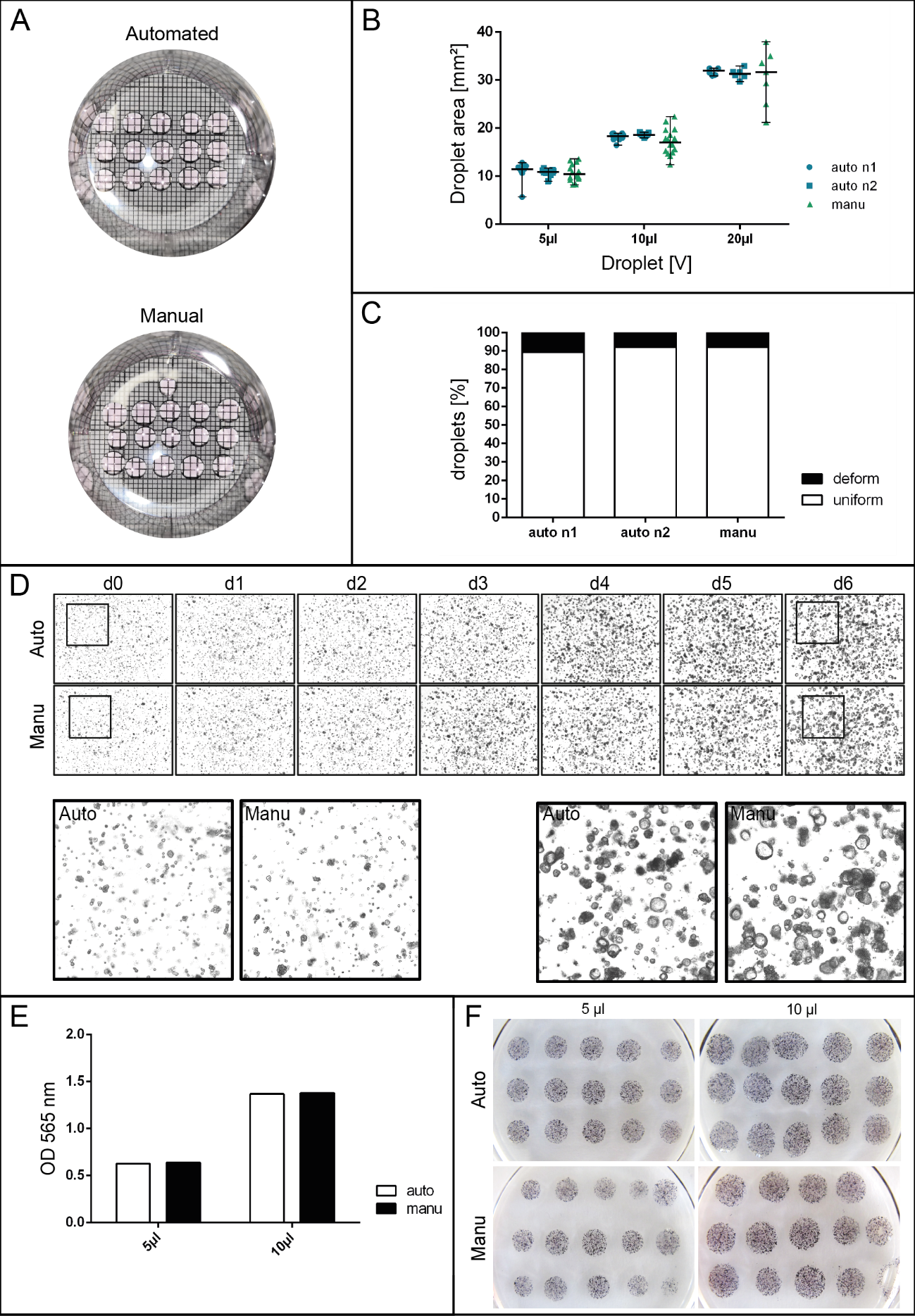


Supplementary Figure 5. Proof of concept Matrigel and organoid handling.

**A.** Macroscopic analysis of drop formation. **B.** Droplet area analysis. Whiskers indicate minimum and maximum values. **C.** Percentage distribution of the droplet shape. **D.** Comparison of organoid growth after manual or automated distribution. **E.** Cell viability (MTT) after 6 days of culture. **F.** Qualitative organoid distribution after 6 days of culture.

**Supervision of critical processes**


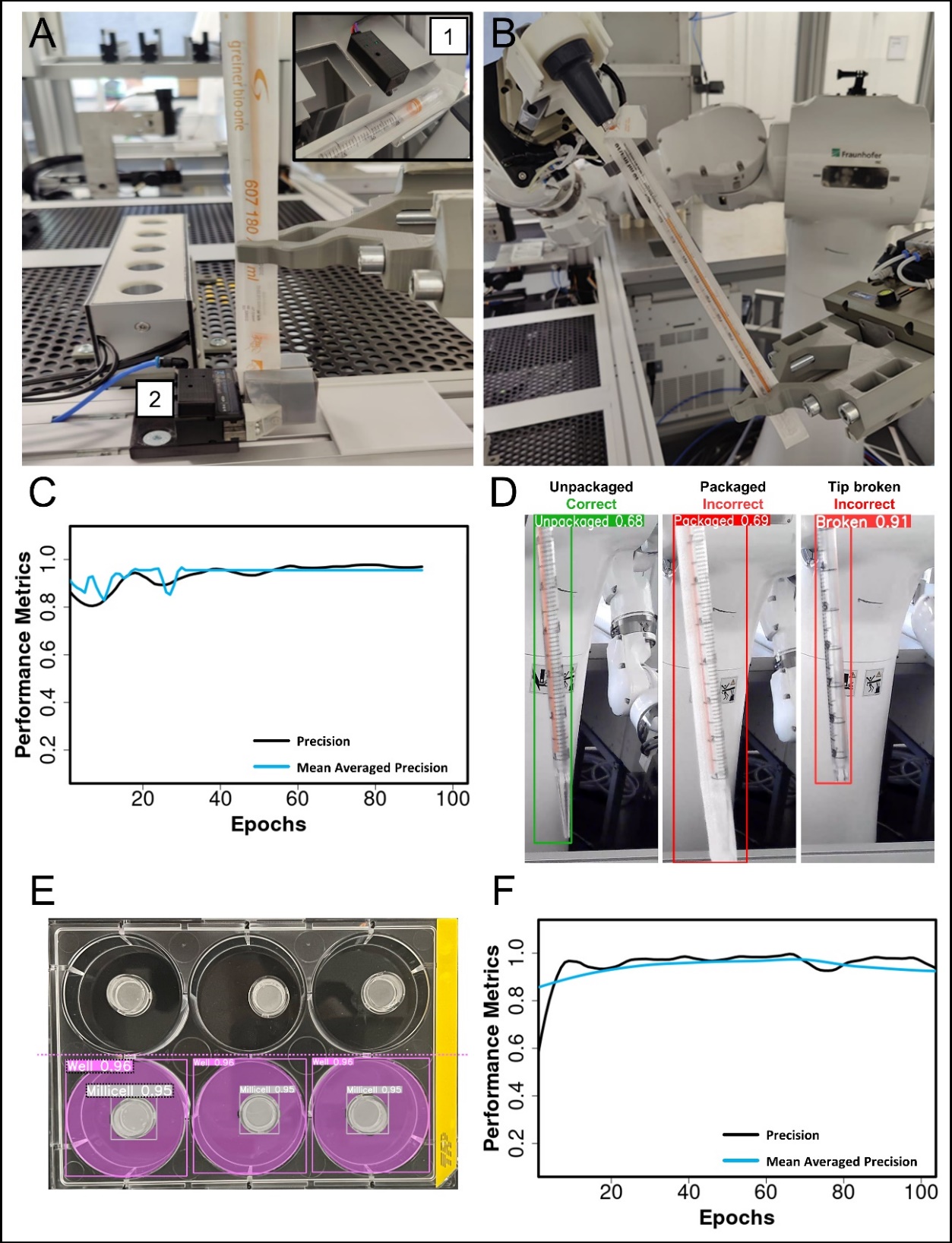


Supplementary Figure 6. Proof of concept pipette unwrapping supervision and insert detection. The robotic workflow included the removal of the sterile wrapping and subsequent insertion of the pipette. Automated pipette supervision was employed to ensure that the packaging was removed successfully and that the graduated pipette was not broken. A. The robotic parallel gripper is handed over the pipette for separation through an inlet rack. An infrared proximity sensor module (1; Tomson Electronics, India) is mounted on this rack to ascertain the presence of a pipette (inset). A slight vibration effect is noticeable when the packaging is torn open. Therefore, a vibration sensor module (2; Funduino GmbH, Germany) is installed to detect this effect. The sensor is mounted via a 3D-printed part to the pneumatic gripping unit, which is specifically designed to hold the sterile wrapping during the opening procedure. B. Next, the pipette is inserted into the silicone attachment. Once mounted to the pipette, the parallel gripper is rearranged to surround the pipette and remove the packaging. C. A YOLOv8 detection model was deployed to verify whether the packaging of the pipette tip has been successfully removed and to ensure that the tip is not broken by the robotic gripper. The training dataset comprises 886 images categorized into three classes: unpackaged tip, packaged tip, and broken tip. The performance metrics graph indicates the class-wise precision and mean average precision (mAP) for all classes over 93 epochs, with a batch size of 64. Precision measures the ratio of true positive detections to the total number of positive detections, providing a direct measure of the accuracy for each prediction. mAP is calculated by averaging the average precision (AP) values, which represent the area under the precision-recall curve for each class, across all classes and multiple recall thresholds. This offers a more comprehensive assessment of the model's overall performance. mAP fluctuates in the early epochs, indicating that the model's ability to balance precision and recall is variable at the start. Precision remains relatively stable, showing consistent accuracy in identifying positive cases. D. A representative example of detection from each class is shown from the test set, which consists of 30 images. The precision for the test set is calculated as follows: 0.969 for the packaged tip class, 0.97 for the unpackaged tip class, and 0.967 for the broken tip class. E. We demonstrate the detection of the positions of the wells and Millicell inserts in a cell culture well plate using a YOLOv8 segmentation model. This detection ensures the correct insertion of pipettes either into the well or the insert, depending on the task being performed. The test set contains 20 images, with the precision for segmentation masks calculated as 0.96 for the wells and 0.83 for the Millicell inserts. F. The training dataset consists of 233 images with two classes: well and Millicell. The performance metrics graph shows the precision and mAP for both classes over 100 epochs, with a batch size of 64. In segmentation, precision refers to the proportion of correctly identified pixels (true positives) among all pixels identified as part of the segment (true positives and false positives). Further, mAP is computed similarly but focuses on the overlap between predicted and actual segments across different thresholds. Precision shows more fluctuations, indicating instability in the accuracy of positive pixel predictions, while mAP follows a smoother trend, reflecting a more consistent overall performance of the model across different thresholds. These observations and results are expected to stabilize with more epochs, a larger dataset, and further fine-tuning of parameters.

# **Additional Supporting Information**

**Supplementary Video 1:** Isolated airway epithelial cells with ciliary beating for airway tissue model construction. The video shows isolated airway epithelial cells on a glass slide by light microscopy. Single cells with ciliary beating indicate viability and functionality and could be used for airway tissue model construction (.mp4).

**Supplementary Video 2:** Beating kinocilia and mucus production of hATM. Primary tissue models matured at the ALI for three weeks until beating kinocilia and mucus production was observed using a light microscopic level and a high-speed camera (.mp4).

**Supplementary Video 3:** Automated conduction of cell culture steps within the ReBiA system. The video shows the interaction of the robot with its automated periphery and tools, such as the automated pipette, and general laboratory materials, e.g. cell culture flasks and Falcon tubes (.mp4).

**Supplementary Video 4:** Physiological barrier formation of hATM. A 3D-reconstruction of immunofluorescence-labeled hATM shows the formation of tight junction marker ZO-1 at the apical surface of airway cells, indicating a physiological cell polarization and the formation of a barrier function (.mp4).

**Supplementary Video 5:** Real-time machine vision-based recognition of the position of culture inserts within a 6-well plate. The annotated machine-view is displayed on the human machine interface of the ReBiA plant (.avi).

**Supplementary Table 1:** Performing different cell culture processes requires the source and target vessels to be changed and the volumes for liquid transport to be adjusted. In order to use programmed processes several times, neutral transfer positions have been defined for all tools, allowing existing jobs to be recombined to perform new tasks. The robot jobs are named according to the scheme shown. The programmer can directly read the peripherals involved and the action that the job implements from the name of the job. The attached table provides an overview of the current job library (.xlsx).

Developed machine learning algorithms, PLC code (TIA portal project) and raw data are available on a GitHub repository. Please contact the author.
